# Nanomaterials in cancer starvation therapy: pioneering advances, therapeutic potential, and clinical challenges

**DOI:** 10.1007/s10555-025-10267-1

**Published:** 2025-05-10

**Authors:** Nam Anh Tran, Shehzahdi S. Moonshi, Alfred K. Lam, Cu Tai Lu, Cong Quang Vu, Satoshi Arai, Hang Thu Ta

**Affiliations:** 1 School of Environment and Science, Griffith University, Nathan, QLD 4111 Australia; 2 School of Medicine and Dentistry, Griffith University, Southport, QLD 4215 Australia; 3 Gold Coast University Hospital, Southport, QLD 4215 Australia; 4 WPI Nano Life Science Institute, Kanazawa University, Kakuma-Machi, Kanazawa, 920-1192 Japan

**Keywords:** Cancer starvation therapy, Multifunctional nanomaterials, Combination of therapies

## Abstract

Gaining significant attention in recent years, starvation therapy based on the blocking nutrients supply to cancer cells via blood occlusion and metabolic interventions is a promisingly novel approach in cancer treatment. However, there are many crucial obstacles to overcome to achieve effective treatment, for example, poor-targeting delivery, cellular hypoxia, adverse effects, and ineffective monotherapy. The starvation-based multitherapy based on multifunctional nanomaterials can narrow these gaps and pave a promising way for future clinical translation. This review focuses on the progression in nanomaterials-mediated muti-therapeutic modalities based on starvation therapy in recent years and therapeutic limitations that prevent their clinical applications. Moreover, unlike previous reviews that focused on a single aspect of the field, this comprehensive review presents a broader perspective on starvation therapy by summarising advancements across its various therapeutic strategies.

## Introduction

Presently, cancer remains the most fatal disease, beginning with aberrant cellular metabolism that triggers uncontrolled proliferation and metastasis of tumours [1–5]. Typically, cancer treatments involve surgery, radiotherapy and chemotherapy aimed to destroy tumours and prevent metastasis to prolong the life expectancy and enhance the patient’s life quality. However, conventional intravenous chemotherapy has several limitations due to poor bioavailability, whereby the therapeutic dose is not achieved as the drug does not reach the target tumour site. Moreover, repetitive intravenous administration leads to the development of drug resistance, which results in poor efficacy and prognosis in patients. Additionally, surgery is often accompanied by lethal risks including bleeding, undesirable side effects, and damage to nearby tissues and organs [1,6,7]. Hence, innovative approaches to overcome barriers related to strategies have been employed, for example, photothermal therapy [8], photodynamic therapy [9], sonodynamic therapy [10], sonothermal therapy [11], chemodynamic therapy [12], immunotherapy [13], hormone therapy [14], and, especially, starvation therapy [15].

Cancer starvation therapy, which targets the tumour’s blood supply to inhibit its growth and survival, has indeed emerged as a promising strategy in cancer treatment. This approach aims to deprive tumours of essential nutrients and oxygen by disrupting angiogenesis, and the formation of new blood vessels which is crucial for tumour nourishment. Various methods, such as using angiogenesis inhibiting agents, vascular disrupting agents, and transarterial chemoembolisation (TACE), have shown potential in limiting tumour growth. Angiogenesis inhibiting agents which prevent the establishment of new blood vessels and vascular disrupting agents which devastate existing vasculature are applied to terminate the blood flow into tumours [16,17]. Without blood supply, cancer cells become malnourished of oxygen and nutrients, resulting in death. Another method is transarterial chemoembolisation (TACE) which integrates the targeted delivery of chemotherapy to tumours and blockage of its blood supply. However, TACE is mainly limited to vascular tumours mainly for hepatocellular carcinoma [18] but also in metastatic colorectal cancer, neuroendocrine tumours, cholangiocarcinoma, and renal cell carcinoma. Metabolic intervention involves deprivation of intra-tumoural oxygen or nutrients such as amino acids [19], glucose [20], and lactate [21] which cancer cells use as a major energy source to survive and proliferate.

While these therapeutic approaches have demonstrated promise in cancer treatment, there are also drawbacks associated with their utilisation, such as low targeting ability as many of these treatments lack the precision to exclusively target tumour vasculature, which then also affects normal blood vessels leading to adverse effects and reduced therapeutic efficacy. Consequently, the lack of specificity may allow the tumour to develop alternative pathways for angiogenesis, leading to the development of drug resistance [22]. Additionally, by disrupting the tumour’s blood supply, these therapies can create a hypoxic environment within the tumour. This paradoxical effect of tumour hypoxia while initially detrimental to tumour growth can also promote the selection of more aggressive cancer cells that are adapted to survive in these conditions, potentially leading to tumour metastasis and resistance to further treatment [22,23].

Moreover, a single treatment based only on starvation therapy cannot provide a considerable result. The combination with the other cancer treatment modalities provides better solutions to overcome limitations and gain optimistic effectiveness [24]. Furthermore, the development of nanomaterials with high-targeted delivery [25] and multifunctional particles [26] opened new opportunities to close the gap and attain a more efficient cancer therapy. These systems enable targeted delivery of multiple therapeutic agents to the tumour site, enhancing efficacy while minimising adverse effects on healthy tissues. Overall, leveraging nanomedicine for cancer starvation therapy holds immense potential to improve patient outcomes by enhancing treatment specificity, and efficacy, and reducing adverse effects associated with conventional therapies. It represents a cutting-edge approach that addresses some of the limitations of current cancer treatment strategies, ultimately offering hope for better management of the disease and improved quality of life for patients.

Although review articles on starvation therapies have been published elsewhere [27,28], the latest one that covered all aspects was in 2019. This comprehensive review aims to present up-to-date approaches and recent efforts in the application of starvation therapies over the past 3 years. We focus on the combination of starvation therapies with multifunctional nanomaterials, providing an overview of fundamental concepts, recent advancements, and key challenges in the development of more effective treatment strategies.

## Conventional approaches of starvation therapy

**Scheme 1:**
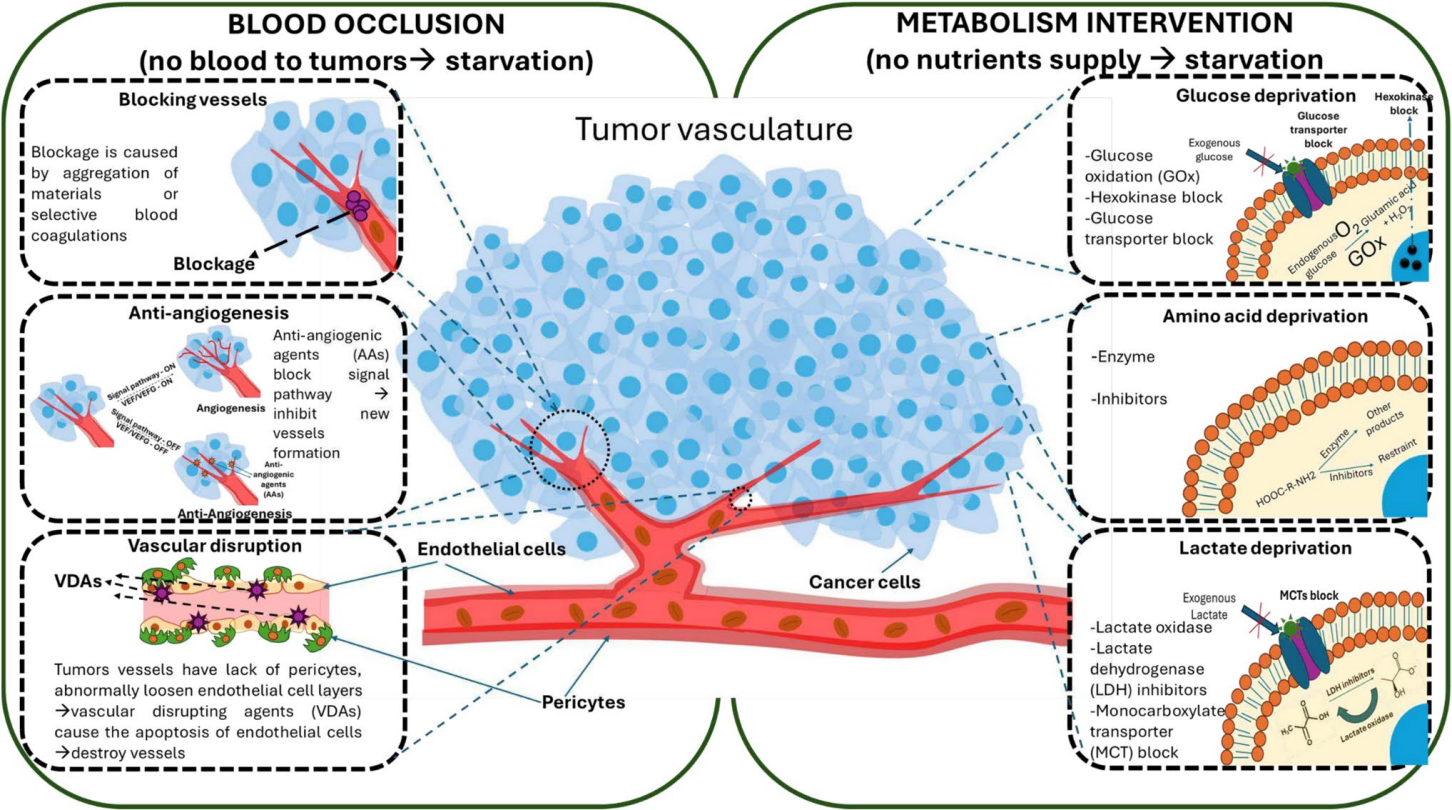
Strategies in starvation therapy. Two main approaches used to induce the starvation include blood occlusion (blocking vessel or embolisation, anti-angiogenesis, vascular disruption) and metabolism intervention (glucose deprivation, amino acid deprivation, lactate deprivation)

Starvation therapy is a comprehensive cancer treatment strategy aimed at suppressing tumour growth and preventing metastasis by cutting off the tumour’s supply of nutrients and oxygen, as well as blocking pathways involved in metastatic spread. Two main targets of this treatment are tumour vasculature and cancer cell metabolism. The first target includes many strategies such as vascular embolisation (blocking vessels), anti-angiogenesis, and vascular disruption. The latter target focuses on metabolic intervention, namely glucose deprivation, amino acid depletion, and lactate deprivation. Scheme 1 summarises the mechanism of these starvation-inducing strategies.

### Angiogenesis inhibiting and vascular disrupting agents

The systemic system plays an important role in the distribution of nutrients and oxygen to every cell in the body. Like normal cells, cancer cells also require them to survive and divide. Moreover, the uncontrolled growth of tumour cells requires an increased supply of nutrients and oxygen. Upon growing beyond a few millimetres in size, tumours release chemical signals (vascular permeability factor (VPF)/vascular endothelial growth factor (VEGF, VEGF-A)) that stimulate the formation of new blood vessels from existing vasculature to satisfy the demand for nutrients and pave the way for metastasis. Some tumours will depend on the existing blood vasculature to nurture [29,30]. Therefore, blocking the blood supply to tumours will effectively restrain the growth and metastasis via the removal of crucial provisions. Two main approaches for blood supply obstruction are the (1) destruction of existing vasculature which is termed a vascular disruption agent and (2) inhibition of new blood vessel formation which is termed an anti-angiogenesis agent. U.S. Food and Drug Administration (FDA) has approved a list of agents for these purposes in cancer treatment that is performed in Table 1.

**Table 1:** Mechanism and agents of angiogenesis inhibition and vascular disruption [31,32]

	Angiogenesis inhibition	Vascular disruption
Mechanism	Binding with angiogenic factors/receptors and inactivating the angiogenesis process	Causing the change in shape of endothelial cells which leads to the decrease in vessel size
Agents	Axitinib, bevacizumab, cabozantinib, everolimus, lenalidomide, lenvatinib mesylate, pazopanib, ramucirumab, regorafenib, sorafenib, sunitinib, thalidomide, vandetanib, ziv-aflibercept	Combretastatin A4 phosphate, AVE8062, ZD6126, ABT-751, MN-029, TZT-1027, DMXAA

Tumour vasculatures have a high proliferation of endothelial cells, and lack of pericytes and the morphology is longer than healthy cells which facilitates the blocking of selective vessels. Moreover, in preclinical and clinical trials, anti-angiogenic agents (AAs) and vascular disrupting agents (VDAs) have demonstrated efficacy in occluding blood supply to tumours resulting in the suppression of its growth. However, the side effects related to anti-angiogenic medicines and the lack of efficacy in preventing multi-factor-mediated angiogenesis are crucial barriers to overcome to obtain therapeutic benefits by this conventional approach [29]. A significant drawback of VDAs treatment is that malignant cells at the outer tumour rim after treatment are still viable. These survival cells could regenerate new tumours with resistance to drugs. Moreover, the cancer cells are highly adaptive. They can modify their metabolism or take nutrients from surrounding tissues or non-damaged blood vessels to maintain their life [33].

### Vascular embolisation (blocking vessels)

One of the valuable targeting strategies for devascularisation is embolisation, in which tumour vasculatures are directly blocked by using embolic agents such as gelatine sponge [34], polyvinyl alcohol nanoparticles [35], Mg_2_Si [36], or enzymes to cause thrombus. This obstruction not only suppresses the proliferation by interrupting the delivery of oxygen and nutrients to tumours but also encumbers metastatic spreading. Instead of using chemical agents to destroy vascular and inactivate angiogenic factors, embolisation usually utilises physical blockade to trigger starvation. Therefore, the therapeutic resistance and toxicity could be minimised. However, the embolisation should be controlled precisely with the highly tumour-selective delivery to prevent unfavourable thrombosis occurring in normal vessels. The clinical application of this approach is transarterial chemoembolisation (TACE), in which the embolic agents are carried precisely to the tumour artery under the support of imagining equipment. Despite good therapeutic results achieved, this technique is restrictively implemented for hepatocellular carcinoma and usually requires an additional surgical operation [37].

The most popular embolic agent is thrombin, an endogenous trypsin-like allosteric serine protease, that triggers the clotting of blood via the regulation of platelet aggregation and promotes the conversion of fibrinogen into insoluble fibrin in plasma. In this way, thrombosis occurs and causes blockage in blood vessels [38].

### Metabolic interventions

#### Mechanism of metabolic processes in cancer cells

Glycolysis is the most vital metabolic process which provides energy for cell activities through breaking down glucose into adenosine triphosphate (ATP) and nicotinamide adenine dinucleotide phosphate hydrogen (NADH). In healthy cells, glucose is converted to 36 molecules of ATP in the presence of oxygen. Conversely, in the acidic and hypoxic microenvironment of cancer, the Warburg effect describes the preference of cancer cells for aerobic glycolysis, in which almost glucose is converted to lactate and only 2 ATP are produced. This process is considerably less efficient than normal oxidative phosphorylation in the production of sufficient energy for maintaining normal life in mammals.

Consequently, cancer cells demand a huge amount of glucose, amino acid, and glutamine to survive due to inefficient energy production [39]. Metabolic interventions such as starvation therapy have been explored as potential adjunct treatments for cancer. By limiting the availability of glucose, amino acids, and other nutrients that cancer cells heavily rely on, the goal is to induce metabolic stress and ultimately trigger apoptosis or necrosis in cancer cells.

#### Glucose deprivation

There are 2 main approaches for glucose consumption inhibition including glucose oxidation into other compounds and inactivation of enzymes catalysing glycolysis. Glucose oxidation–mediated starvation therapy relies on glucose oxidase (GOx), a glycoprotein which has 2 polypeptide chains and 2 adenine dinucleotide coenzymes. GOx catalyses the oxidation of glucose into gluconic acid and by-product H_2_O_2_ under the presence of O_2_. Enhanced conversion into gluconic acid results in the inevitable reduction of cellular glucose concentration which triggers an energy crisis and apoptosis in cancer cells. Nonetheless, intra-tumoural oxygen consumption can worsen hypoxic conditions, and the production of gluconic acid can increase cellular acidity, further promoting tumour survival and progression. Furthermore, the paucity of intracellular oxygen restricted glucose oxidation. This exacerbates the tumour microenvironment and weakens therapeutic effectiveness. The accumulation of by-product H_2_O_2_ augments the oxidative stress and cytotoxicity, potentially harming surrounding healthy tissues. Moreover, glucose oxidation can occur in the bloodstream, reducing the delivery of GOx to cancer cells and hindering its efficacy. This approach has shown potential in preclinical studies, but their translation into effective clinical therapies faces challenges such as systemic toxicity, off-target effects, and limited efficacy [39,40]. Combining these approaches with other treatments or developing more targeted delivery methods may improve their clinical utility.

Some medicines, such as 3-bromopyruvate [41], lonidamine, and its derivatives [42], interfere in glycolysis by blocking hexokinase in mitochondria. However, this blockage occurs temporarily and is reversible. The therapeutic efficacy of a single-use lonidamine is usually low and not enough to suppress the proliferation of cancer cells. Moreover, the hydrophobic and low mitochondria targeting also hinder the employment of lonidamine in oncology treatments [43].

#### Amino acid depletion

Tumours often exhibit an increased demand for exogenous amino acids from the bloodstream to support their rapid growth and proliferation due to the inefficient utilisation of glucose for energy production. The uptake of amino acids by cancer cells is tightly regulated by cellular signalling pathways, notably the mammalian target of rapamycin complex 1 (mTORC1) and general control nonderepressible 2 (GCN2) pathways. mTORC1 plays a central role in promoting cell growth and proliferation in response to nutrient availability, including amino acids. GCN2 is activated in response to amino acid deprivation or starvation, leading to cellular responses aimed at conserving energy and promoting survival. When amino acid availability is limited for an extended period, cells activate stress response pathways such as GCN2, which suppresses mTORC1 activity. This response helps cells adapt to nutrient scarcity and maintain viability. However, prolonged amino acid deprivation can also trigger apoptosis, leading to cell death [44]. Targeting amino acid metabolism and the signalling pathways involved in amino acid sensing and utilisation represents a promising approach for cancer therapy. By disrupting the balance of amino acid supply and demand in cancer cells, these interventions listed in Table 2 aim to impede tumour growth and progression [19,45,46].

**Table 2:** Mechanisms and limitations of amino acids depletion therapies [44] [47] [48]

Amino acid	Role in cancer cells	Mechanism of interventions	Limitations	Preclinical and clinical
Glutamine	-Most consumed nutrients (next to glucose) -Important for the tricarboxylic acid cycle -Utilised in almost syntheses of nonessential amino acid	-Glutaminase increases glutamine synthesis when enhancing glutaminolysis -Inhibition of Glutaminase by inhibitor: CD-839, BPTES	-Development of drug-resistant mutations toward inhibitors	Yes
Asparagine	-Stimulated glutamine biogenesis leading to epithelial to mesenchymal transition which drives metastasis	-Using asparaginase (ASNase) to restraint biosynthesis -ASNase can also prohibit glutamine synthesis	-Most successful amino acid depletion therapy -Therapeutic resistance -Cancer cells can alternatively utilise glutamine	Yes
Arginine	-Important role in stabilisation of proteins -Precursor for active compounds for metastasis and DNA damage	-Depletion caused by enzymes human arginase or the bacterial arginine deiminase which converts arginine to ornithine or citrulline	Therapeutic resistance	Yes
Methionine	-Active role in malignant transformation	-Isolating cancer cells from exogenous methionine supply -L-methionine-gamma-lyase converted methionine to the other products	Therapeutic resistance	Yes
Serine and cysteine	-Crucial role in proteins, phospholipids and glycine synthesis, -Participate in the folate cycle for producing nucleotides	-Using cyst(e)inase and phosphoglycerate dehydrogenase inhibitors combined with dietary restriction	Therapeutic resistance	Preclinical only

Amino acid depletion therapies may lack specificity, leading to off-target effects and potential toxicity to healthy tissues. Amino acids are essential for the function and regulation of immune cells, including those involved in anti-tumour immunity. Depleting amino acids indiscriminately may impair the immune response against cancer, potentially compromising the effectiveness of immunotherapy strategies. Moreover, tumours exhibit considerable heterogeneity in their metabolic profiles, even within the same type of cancer. Additionally, cancer cells can adapt to nutrient limitations by altering their metabolic pathways or acquiring nutrients through alternative mechanisms. These adaptations may reduce the effectiveness of amino acid depletion therapy and contribute to treatment resistance. Hence, improving the delivery and specificity of inhibitors is essential to minimise adverse effects ^[[40]]^.

#### Lactate deprivation

While lactate was traditionally viewed as a waste product of glycolysis, recent research has shown that cancer cells can utilise lactate as a significant energy source, particularly under conditions of glucose deprivation or hypoxia. This metabolic adaptation allows cancer cells to survive and proliferate in nutrient-limited environments. Lactate not only sustains cancer cell survival but also influences the tumour microenvironment in ways that promote metastasis and angiogenesis. Intra-tumoural lactate concentration can be limited by therapeutic approaches including restricting lactate production, inactivating transporter, neutralisation, and lactate oxidation. Lactate dehydrogenase (LDH) inhibitors such as oxamate, gossypol, and PSTMB can block the conversion of pyruvate to lactate, thereby reducing lactate production in cancer cells. Monocarboxylate transporters (MCTs) facilitate the uptake and efflux of lactate from cancer cells. Inhibiting MCTs can limit lactate export from cancer cells, potentially mitigating its effects on surrounding tissues and immunity. Therapeutic strategies that neutralise extracellular lactate or promote its oxidation within cancer cells are also being explored as potential treatments to reduce lactate levels and disrupt cancer metabolism [49–52].

## Cancer starvation therapy based on nanomaterials

### Nanomaterials as starvation agents–blood occlusion and metabolism intervention

Nanomaterials themselves can possess properties that cause starvation via blood occlusion and metabolic interventions.

Gold and silver nanoparticles can block signalling pathways or inhibit the secretion of pro-angiogenic factors, complementing the action of loaded anti-angiogenic compounds. Several nanomaterial-based formulations have shown promise in inhibiting angiogenesis and suppressing tumour growth. Zinc, titanium, selenium, sulphur, and cerium compounds exhibit dual properties, either angiogenesis or anti-angiogenesis depending on the compounds consisting of them and the surrounding environment. For example, cerium oxide’s dual behaviour in angiogenesis was affected by pH, ROS level, and concentration of nanoparticles [53]. Selenium-gold nanostructure [54] and Elaeagnus angustifolia L-Fe_2_ZnO_4_ [55] are promising anti-angiogenic particles which successfully interfered with the VEFG/VEFGA.

**Fig. 1:**
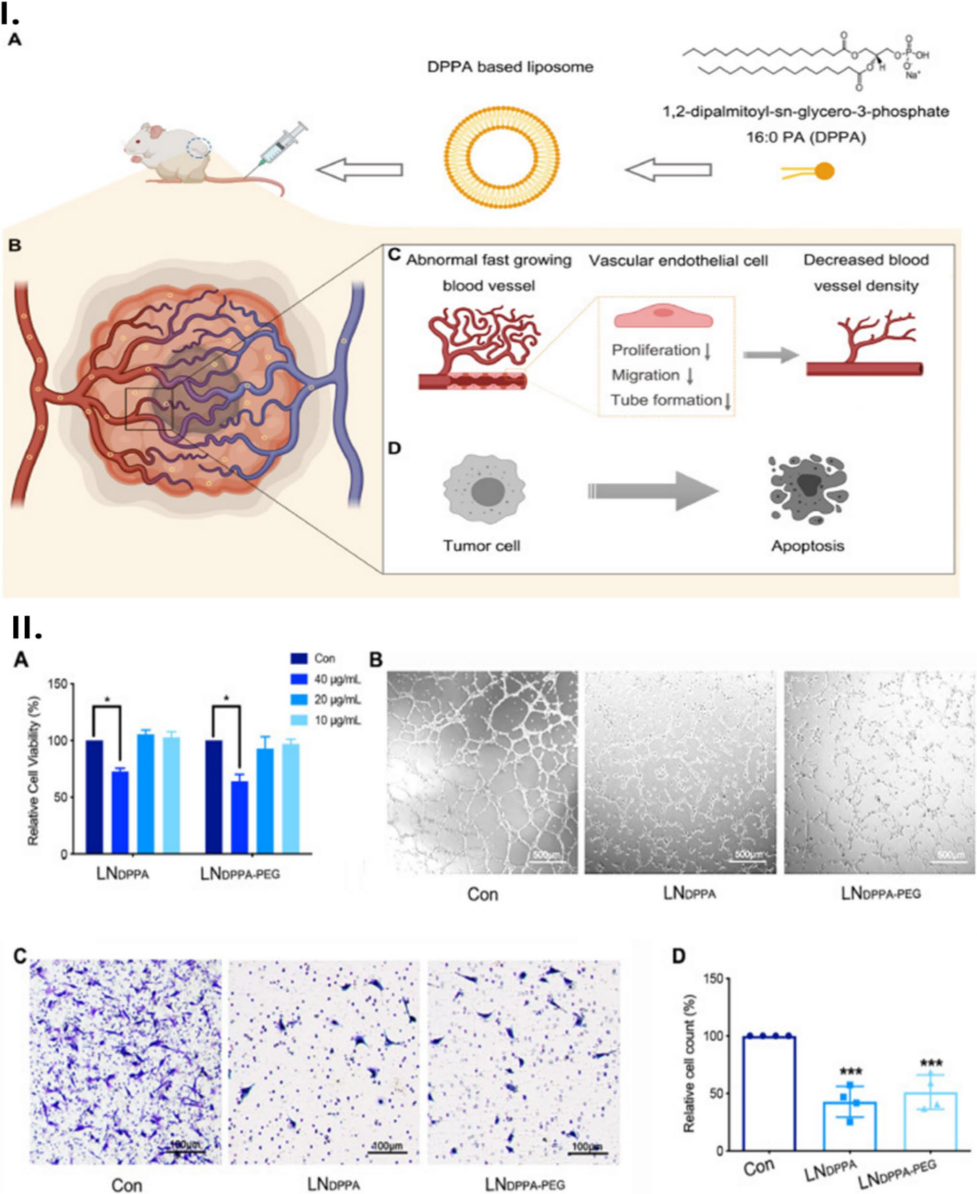
**I** Schematic illustration of the anti-tumour and anti-angiogenic effect of DPPA-LNP. (**A**) The bioactive lipid DPPA was utilised to prepare DPPA-LNP. (**B**) DPPA-LNP efficiently accumulated in the tumour area after intravenous injection. In TME, DPPA-LNPs achieve an anti-angiogenic effect by inhibiting vascular endothelial cell proliferation, migration, and tube formation (**C**); meanwhile, it directly induces tumour cell apoptosis (**D**). **II** *In vitro* anti-angiogenic effect of DPPA-LNPs. (**A**) The HUVEC viability after treated with indicated concentration of LNDPPA or LNDPPA-PEG for 24 h; (**B**) the HUVEC tube formation inhibition ability of DPPA-LNPs after co-incubated HUVEC with 20 µg/mL LNDPPA or LNDPPA-PEG for 6 h (scale bar indicated 500 µm); (**C**) the HUVEC migration inhibition ability of DPPA-LNPs after co-incubated HUVEC with 20 µg/mL LNDPPA or LNDPPA-PEG for 12 h (scale bar indicated 100 µm); (**D**) the quantification of migrated HUVEC of transwell assay (*p < 0.05; ***p < 0.001 compared with Con group) [56]. Reproduced with permission. Copyright 2023, Elsevier

An anionic phospholipid (dipalmitoyl phosphatidic acid, DPPA) was prepared as lipid nanoparticles via the precipitation method and employed as an anti-angiogenic agent which not only inactivated Homeobox cut like 1 (CUX1)/fibroblast growth factor 1 (FGF1) hepatocyte growth factor (HGF) signalling pathway but also successfully restricted the growth of breast cancer (4 T1 cells) (Fig. 1). The DPPA liposomal nanoparticles (DPPA-LNPs) overcame the barriers of the conventional use of DPPA which were superhydrophobicity and side effects. In the form of nanoparticles, the anti-angiogenic and anti-tumoural properties of DPPA were retained and the tumour-targeting delivery was improved considerably. The DPPA-LNPs concentration of 20 µg/mL not only impeded notably the tube formation of human umbilical vein endothelial cells (HUVEC) but also suppressed the migration of these cells by around 50%. At the higher concentration of these liposomal nanoparticles (40 µg/mL), the HUVEC proliferation was constrained significantly [56].

Polyphenol nanoparticles, which were formulated via the coordination of iron and 15 polyphenols respectively, performed not only notable vascular disruption but also excellent anti-angiogenesis. These polyphenols naturally have high hydrophobicity and poor solubility despite their good inhibition of new vessel development via the preferential binding to VEGFR2 (vascular endothelial growth factor receptor 2). Therefore, their translation for clinical applications usually was obstructed. The respective assembly of 15 polyphenols and iron is a powerful approach to overcome limitations. Naringenin, hesperidin, catechin, quercetin, silybin, ellagic acid, curcumin, myricetin, luteolin, morin, caffeic acid, chrysin, gallic acid, dopamine, and EGCG spherical shape nanoparticles (diameter from 2 to 150 nm) have shown their anti-angiogenic activity. At concentrations of 200 µg/mL, polyphenol nanoparticles consisting of ellagic acid, gallic acid, and quercetin provided preponderant new vessel suppression and selectively vascular interruption for the treatment of high-grade glioma [57].

**Fig. 2:**
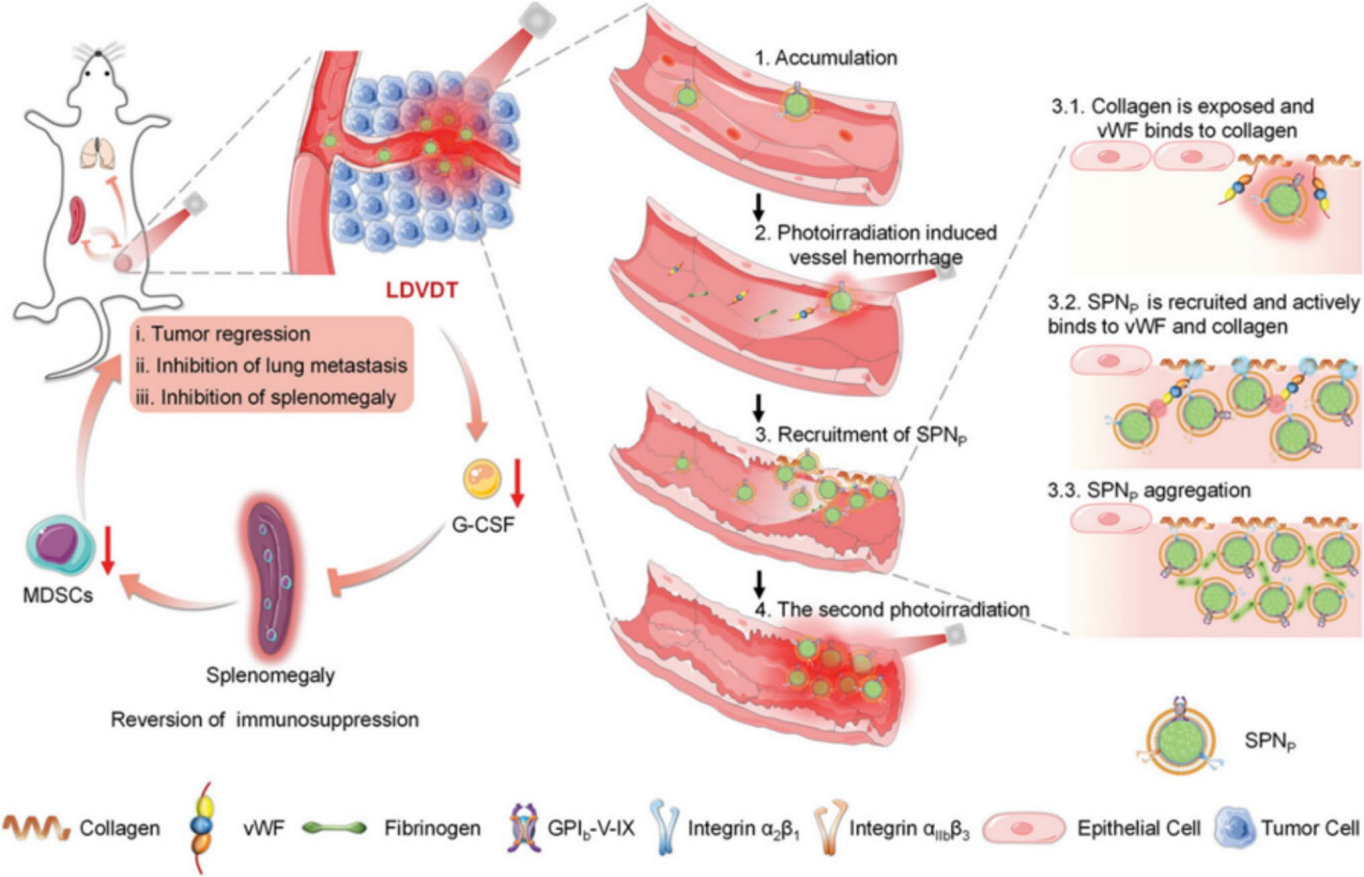
Schematic illustration of light-driven vascular targeting and disruption therapy (LDVDT) using polymer nanoparticles. The 808 nm irradiation triggered mild hyperthermia that caused the tumour vascular haemorrhagic damage and activated the coagulation cascade. After that, the damage generates collagen exposure and uncoils the von Willebrand factor (vWF) in plasma. This facilitated the accumulation of nanoparticles through their binding to membrane proteins GP1b-V-IX and integrin α2β1. Finally, nanoparticles aggregated and enhanced hyperthermia significantly to not only destroy vessels but also eradicate tumours [59]. Reproduced with permission. Copyright 2023, Wiley Periodicals LLC

Instead of merely changing endothelial cells’ shape, recent approaches employed nanoparticles to trigger vasculature damage with heat produced via photothermal treatment therapy. The localised heating of the tumour vasculature leads to several effects, including endothelial cell damage, vessel coagulation, and disruption of blood flow. This can result in vascular occlusion, ischemia, and ultimately, tumour necrosis. 5,6-Dimethylxanthenone-4-acetic acid (DMXAA)–mediated fibrinogen-conjugated AuNPs aggregation amplified the photothermal-supported tumour vascular disruption [58]. A combination of semiconducting polymer nanoparticles, which produced heat under near-infrared irradiation, with platelet membranes for activatable vascular targeting, provided novel nano-sized systems for light-driven vascular targeting and disruption therapy (LDVDT). The generated heat-engendered vascular disruption enhanced the activation of coagulation cascades and recruited the blood circulation of polymer nanoparticles toward injured vessels. This improved the targeting delivery to the tumour region. At the dosage of 200 µL (concentration of polymer of 150 µg/mL), this system could not only eradicate the tumour utterly but inhibited the metastasis of lung cancer (4 T1 cells) remarkably under 808 nm laser irradiation (0.3 W/cm^2^) (Fig. 2) [59].

Predominantly, metabolic interventions are based on the utilisation of enzymes, especially glucose oxidase (GOx) to catalyse the transformation of glucose. These enzymes usually were loaded on nanocarriers to improve the target transportation via the covalent conjugation or electrostatic interactions between enzymes and nanomaterials. Despite the notable refinement of selective targets to tumour tissues, there are many limitations, for example, leaching of enzymes, aggregation of nanocarriers, and limited loading capacity hinder the clinical translations. The ideas exerting alternatives relied on enzyme-mimicking materials such as metal nanoparticles, especially ultrasmall Au nanoparticles, which are auspicious solutions for these problems. Moreover, the acidity of the tumour microenvironment also prevents effective metabolic interventions via triggering cell cycles to induce autophagy that improves the survival of cancer cells under a starvation state [60]. Calcium phosphate (CaP)–coated Au nanocomposites loaded with MCT4 inhibitor fluvastatin mimicked the glucose oxidase (Au nanoparticles) and restricted autophagy by blocking cellular efflux of lactate using an MCT4 inhibitor (fluvastatin)[60]. Au@BSA-L-wzb117 was reported as a multifunctional system where Au catalysed glucose oxidation and wzb117 inhibited the glucose transporter 1 (GLUT1) to prevent the entry of extracellular glucose. This two-pronged strategy was highly selective to the tumour region via the hypoxia-triggered release of wzb117 by breaking hypoxia-responsive linker 4,4-azodibenzoic acid; therefore, the tumour growth was suppressed strikingly but not eradicated [61]. Cobalt nanoparticles were utilised for dual amino acids and oxygen depletion therapy, in which, cobalt nanoparticles formed the complexes with amino acids to carry oxygen molecules and oxidise them. The simultaneous deprivation presented positive effects on killing cancer cells [62]. During blood circulation, the interactions between these nanomaterials and nutrients in blood could cause adverse effects such as blood sugar level drop and deprivation of amino acids that are necessary for normal tissues to survive. Moreover, low targeting delivery and undesirable leakage of active compounds during vascular transport could lead to the risk of intoxication. The tumour-responsive strategies should be considered carefully to improve the safety of therapies and prevent severe effects on normal cells.

### Nanomaterials as carriers for targeted delivery of starvation-inducing agents

Nanomaterials offer precise delivery of starvation-inducing compounds to aberrant cells while protecting healthy tissues, thus reducing side effects. Their nanostructure enables them to penetrate biological barriers more effectively. Furthermore, the use of nanocarriers allows for controlled release of active compounds, prolonging their pharmacological activity, and enhancing selective cellular accumulation. This controlled release is particularly beneficial for maintaining therapeutic efficacy over a long period.

Various nanomaterials are utilised as delivery systems for *anti-angiogenic agents*, including polymers (e.g. PEG, PLA, PLGA), liposomes, and large-surfaced carbon-based nanomaterials (e.g. graphene oxide, carbon nanotubes, nanodiamonds). These materials are chosen based on their compatibility with anti-angiogenic medicines (AAs) and their ability to efficiently deliver drugs to target sites [63–65]. Graphene oxide nanoparticles containing 6-gingerol successfully suppressed the expression of glutathione peroxidase (GPx), superoxide dismutase (SOD) antioxidant enzymes, VEGF, and VEGF-R genes in gastric tumour cell lines at a concentration range of 26–36 µg/mL [66]. RGD1-R6 peptide–carrying siRNA nanoparticles [67], metformin-loaded gold-poly(catechin) core–shell nanoparticles [68], and low-density lipoprotein (LDL) nanosystem encapsulated Vandentanib [69] are promising anti-angiogenic particles which successfully interfered with the VEFG/VEFGA.

Cardiotoxicity and short half-life are Achilles’ heels of conventional *vascular disrupting agents (VDAs).* Therefore, nano-sized carriers are substantially helpful for targeting the delivery of VDAs to tumours and the improvement of cellular accumulation of VDAs. Usually, monotherapies of only one of the VDAs or AAs have limited response because of the resistance of tumours. Therefore, the coalition of VDAs and AAs concurrently is a prominent solution to overcome the bottlenecks of monotherapy including drug resistance and tumour recurrence. Platelet membrane–coated mesoporous silica nanoparticle (MSN) co-delivered Combretastatin A4 (CA4) and Apatinib provided the remarkable suppression MHCC-97H liver tumours growth after 25 days. Platelet membrane is ideal for protecting nanocarrier from blood clearance and the immune system. Hence, the transportation of starvation agents is improved undoubtedly [70]. 

The main hindrance to effectual metabolic-intervened starvation therapy is the poor-targeting delivery of *metabolic interruption-inducing compounds*. Instead of tumours, starvation can have adverse effects on normal cells. For these reasons, the conventional approaches did not provide significant benefits in tumour eradication. Nanoparticles have opened new avenues for improving the efficacy and specificity of starvation therapies in cancer treatment. Nanoplatform based on amorphous calcium phosphate (ACP) nano-substrates loaded with metformin and GOx was developed to combine glucose starvation and sensitised metformin therapy [71]. Iridium/ruthenium (IrRu) ultrasmall nanoparticles modified with GOx and PEG improve glucose oxidation by catalysing the decomposition of H_2_O_2_ into O_2_. Moreover, this system also enhanced the formation of singlet oxygen ^1^O_2_ causing apoptosis of cancer cells [72]. Transgenic microorganism Escherichia coli MG1655 (EcM-GDH) microbes that produce glucose dehydrogenase and have a high affinity to tumours were used to initiate apoptosis by depriving glucose nutrition in colorectal tumours [73]. Hyaluronic acid (HA)–functionalised redox-responsive micellar nanosystem encapsulated Lonidamine and (5-phenylacetmido-1,2,4-thiadiazol-2-yl) ethyl sulfide [74], and functional MOF-based core/shell nanoreactor–loaded inhibitors [61,75] respectively provided dual-blocking starvation therapy which restricted not only glycolysis but glutamine metabolism as well. Fluvastatin sodium–, metformin-, and bupivacaine-loaded ClO_2_@CaSiO_3_@MnO_2_-arginine-glycine-aspatic acid nanoparticles were administered to trigger deficiency of methionine via the release of ClO_2_^−^ that oxidised methionine. Moreover, fluvastatin inhibited the MCT4 expression and metformin suppressed the TCA cycle simultaneously [76]. Zeolitic imidazolate framework-8 (ZIF-8) nanoplatforms loaded with α-cyano-4-hydroxycinnamate (CHC) and glucose oxidase (GOx) were employed to trigger the dual deprivation of glucose and lactate hence the efficacy in killing tumours was enhanced considerably when compared with mono-blocking approach [77]. Instead of blocking the lactate production, lactate oxidase was loaded in mesoporous silica combined with mitochondria-targeting drugs to deplete the existing lactate and dysfunctional the mitochondria [78].

**Fig. 3:**
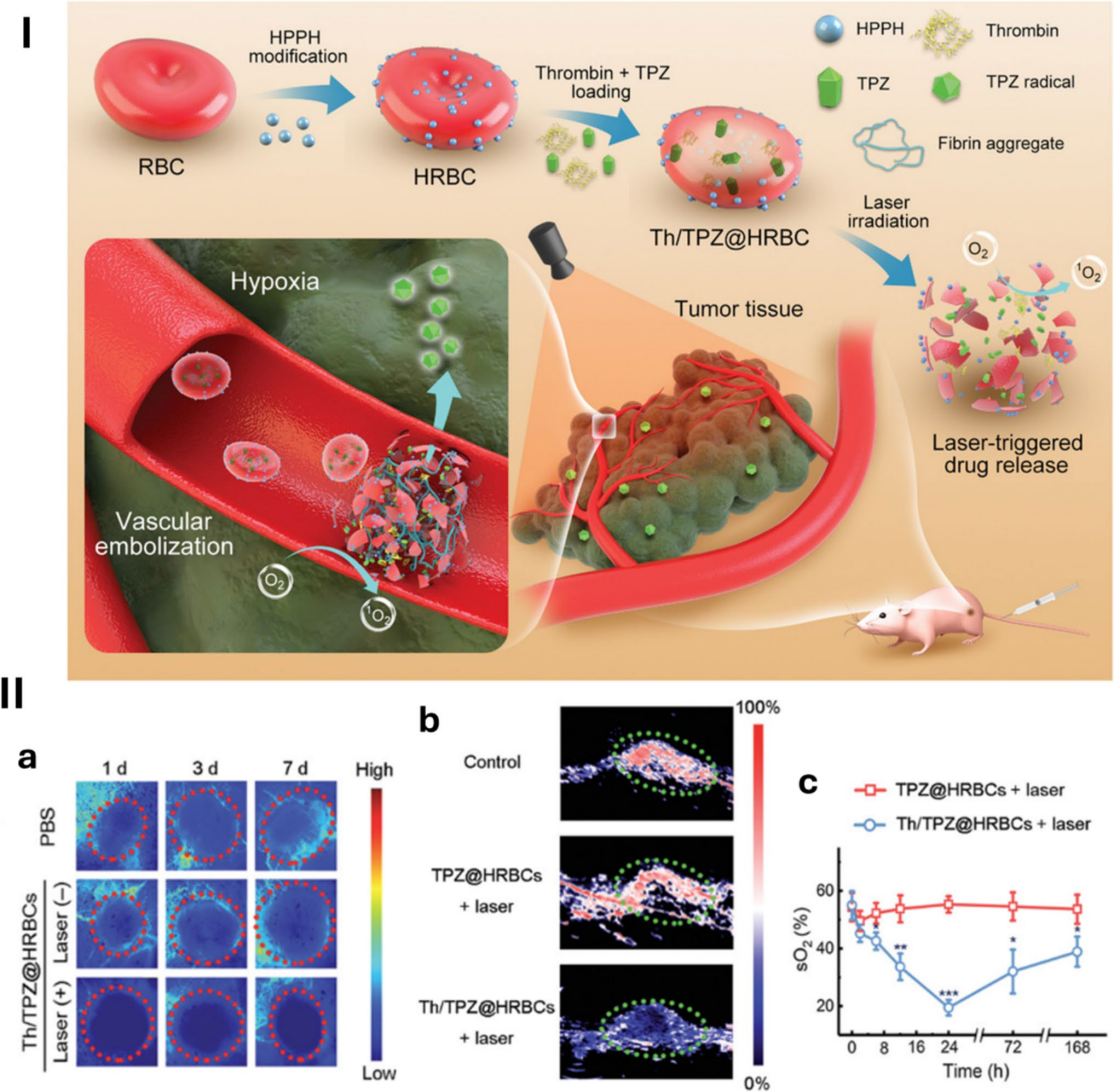
“Photoactivatable bomb” for vascular embolisation. **I** Scheme illustrating the fabrication of Th/TPZ@HRBCs and their applications in laser-triggered tumour vessel blockage and hypoxia-activated chemotherapy. **II** (**a**) Representative colour-coded laser speckle images of the tumour sites in the mice from different groups. Tumour-bearing mice were intravenously injected with PBS (control) or Th/TPZ@HRBCs (thrombin = 500 U kg^−1^, TPZ = 3 mg kg^−1^), and then imaged at 1, 3, and 7 days postinjection, respectively. For the “Laser (+)” group, laser irradiation (671 nm, 30 mW). (**b**) PAI data reflecting the blood oxygen saturation levels of the tumour areas in the 4 T1 tumour-bearing mice intravenously injected with TPZ@HRBCs or Th/TPZ@HRBCs (thrombin = 500 U kg^−1^, TPZ = 3 mg kg^−1^). Laser irradiation (671 nm, 30 mW cm^−2^, 20 min) was carried out at 6 h postinjection. The images were taken at 24 h after laser irradiation. Untreated mice were set as the control group. The green dotted circles indicate tumour regions. Red and blue colours indicate higher and lower blood flow, respectively. The red dotted circles indicate tumour regions. (**c**) Quantified oxygen saturation levels of the tumour area in the mice at different time points after the indicated treatments. Statistical data are presented as mean ± standard deviation (*n* = 5) and the differences between the two groups were analysed by Student’s *t*-test (*p < 0.05, **p < 0.01, ***p < 0.001) [37]. Reproduced with permission. Copyright 2021, Wiley Periodicals LLC

Immediately inducing blood coagulation when contacting directly blood in vessels, embolic agents such as thrombin cannot be directly intravenously injected. Blood clot formation caused by these agents during circulation could significantly reduce the blood flow to normal tissues and cause insufficient vascular blockage at tumour sites. Nanocarriers are the optimal choice for tumour-specific releasing and inducing thrombosis-based starvation. Organic phase-change materials (PCM) co-loaded thrombin (Thr) and IR780 have been constructed as thermal-responsive nanoplatform for controllable-released embolisation at the tumour site. Under 808 nm irradiation, PCM nanoparticles started melting and releasing Thr because of the thermal effect induced by IR780 [79]. Red blood cells (RBC) were decorated with photoactivable 2-(1-hexyloxyethyl)−2-devinyl pyropheophorbide-$\alpha \left(\text{HPPH}\right)$ and co-loaded Thr and tirapazamine (TPZ) to synthesise photoactivable bomb for laser-triggered thrombin release starvation. RBC provided hemocompatibility and improved circulation time of embolic agent and hypoxia-responsive chemodrug during transportation to the tumour site. Under laser irradiation, HPPA generated ^1^O_2_ that burst the RBC to release loaded active drugs in tumour vasculature in a precise and highly controllable way (Fig. 3) [37]. ZIF-8 encapsulated Doxorubicin (DOX) and Thr has been used as a tumour microenvironment-responsive transporter for combined chemoembolisation therapy on 4 T1 cells [80].

### Nanomaterial-based combination therapy

**Scheme 2:**
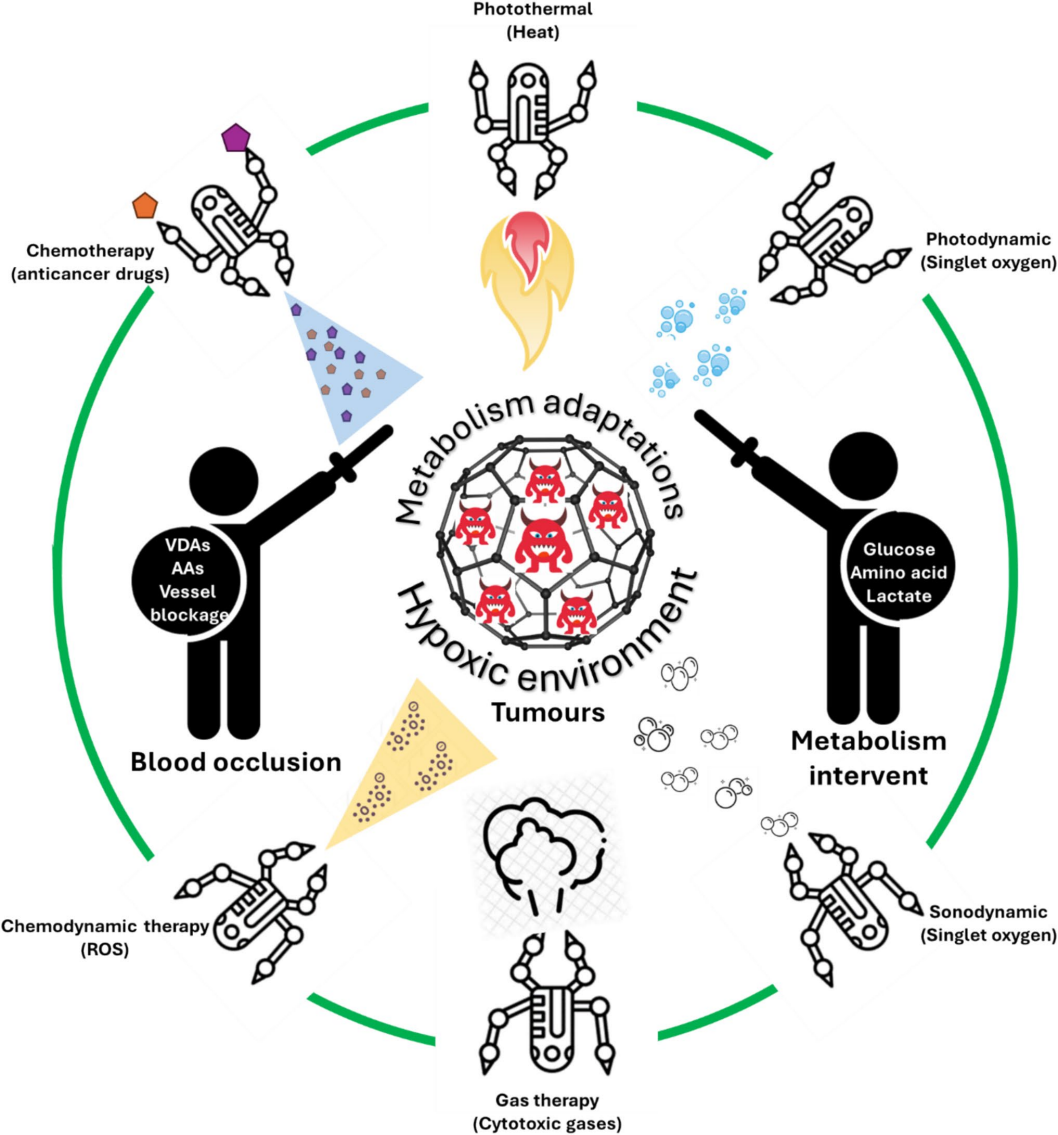
Multi-therapeutic modalities based on starvation therapy

Monotherapy based solely on cellular starvation often proves to be ineffective or only mildly effective in suppressing cancer progression. The adaptability of cancer cells through metabolic modifications is a significant factor contributing to therapeutic failures. Hypoxic conditions in cancer cells activated the hypoxia-inducible factors (HIFs) that orchestrated metabolic response to promote survival and resistance to treatments [81]. The concept of exploiting nutrient scarcity to enhance the vulnerability of cancer cells has gained significant attention in recent years. This vulnerability can be leveraged to improve the efficacy of other treatments, making combination therapy (Scheme 2) a critical approach in cancer treatment.

#### Starvation/chemodynamic therapy

**Scheme 3:**
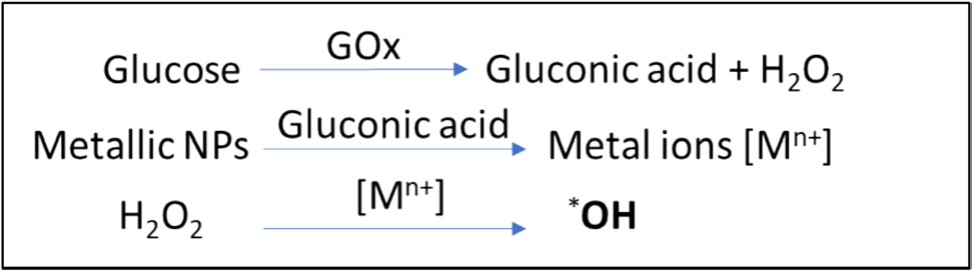
Mechanism of integrated starvation/chemodynamic therapy

H_2_O_2_, a by-product of glucose oxidation, becomes useful for triggering the chemodynamic therapy (CDT) via conversion into *OH, hence enhancing therapeutic efficacy synergistically. OH* radical reacts with cellular components, causes genetic damage, and triggers cell apoptosis via activation of caspases while glucose starvation caused by glucose oxidation can disrupt protein interaction that leads to loss of mitochondrial membrane potential and ultimately triggers the cell apoptosis [82,83]. This combined attack enhances the effectiveness of killing cancer cells. Moreover, the formation of gluconic acid also assists glutathione (GSH) in facilitating the release of metal ions from nanoparticles such as Fe^2+^ and Fe^3^, to catalyse the Fenton process (Scheme 3). This combination is a key to figuring out not only the increase of cellular acidity but also the cytotoxicity of H_2_O_2_.

The starvation caused by glycolysis inhibition via blocking transporters will not produce H_2_O_2_. In this case, metal ions can be exerted to decompose endogenous H_2_O_2_, hence stimulating dual treatment. Because of the low concentration of endogenous H_2_O_2_, the therapeutic effectiveness will not be as good as the glucose oxidation/chemodynamic approach. This dual strategy has been adopted successfully in various reported studies for many cancer cell types (Table 3).

**Table 3:** Representative recent efforts in nanomaterials supported starvation/chemodynamic therapies

Nanomaterials	Metallic ions	Starvation causing agents	Cell lines	Duration of *in vivo* treatment (days)	Tumour suppression (S) or eradication (E)	Ref
Janus c-Fe_2_O_3_/SiO_2_ conjugated GOx	Fe^2^ +/Fe^3+^	GOx	4 T1	15	S	[84]
LipoCaO_2_/Fe(OH)_3_-GOx			MDA-MB-231	16	S	[85]
Fe_3_O_4_@MIL-100@GOx			4 T1	14	S	[86]
ZIF8-loaded GOx, haemoglobin and methemoglobin			4 T1	18	S	[87]
FeGdNP-ICG/GOx-RGD2-mPEG			MKN45 GC	15	S	[88]
ZIF-8/PdCuAu/GOx@HA			T24	28	S	[89]
Iron-based NMIL11			4 T1	14	S	[90]
AS/GOD@HAZnO NPs			4 T1	10	S	[91]
MnSiO3@Met@GOx	Mn^2+^	GOx	4 T1	14	E	[92]
F127–MnO2-ZIF-8@GOx			HeLa 4 T1	No *in vivo*	No *in vivo*	[93]
GOx with manganese-doped calcium phosphate (MnCaP)			IDH1 (R132H)	60	S	[94]
Mn_3_O_4_@PDOMs-GOD			SMMC-7721	12	S	[95]
Mn-TCPP = 5, 10, 15, 20-tetrakis (4-carboxyphenyl) porphyrinato-manganese (II) chloride) loaded with GOx			4 T1 A549	14 (4 T1 tumours)	S	[96]
Bismuth − manganese-based nanozyme loaded with GOx			4 T1	14	S	[97]
GOx@MnCoMOF			Cal-27	17	S	[98]
CaCO_3_@MnO_2_-NH_2_@GOx@PVP			4 T1	14	S	[99]
Triptolide (TP) and 2-deoxy-D-glucose (2-DG) loaded into hollow mesoporous MnO2		2-DG	A549	14	E	[100]
Cu2 + -inserted hollow mesoporous silica nanoparticles–loaded GOx	Cu^2+^	GOx	MCF-7	16	S	[101]
ZnO_2_@Au@ZIF-67 NPs	Co^2+^/Co^3+^	Au	4 T1	15	S	[102]
Pd@Pt-GOx/HA	Pd@Pt	GOx	4 T1	15	S	[103]
PCN-224(Cu)-GOD@MnO_2_ nMOFs	Cu^+^/Mn^2+^	GOx	HeLa	14	S	[104]

The pivotal advantage of this combinatory strategy is that the chemodynamic can be triggered without the requirement of external energy such as ultrasound or light. Therefore, the depth of tumour location is no longer a limitation. Generally, the CDT primarily relied on iron and manganese ions as catalysts to generate radicals. Other metal ions, such as Cu^2+^ and Co^2+^, as well as noble metals like platinum and palladium were also explored to undergo the Fenton process. The starvation in this dual strategy was mainly caused by the glucose oxidation using GOx or GOx-mimicking nanoparticles (Au NPs) as catalysts. The utilisation of glycolysis inhibitors in this approach is limited because they cannot produce H_2_O_2_, a crucible factor of CDT. Despite impressive results achieved in cancer treatment, this therapeutic combination possessed certain drawbacks involving the undesirable glutathione (GSH) oxidation catalysed by GOx under the presence of oxygen to produce glutathione disulfide (GSSG). This oxidation leads to an insufficient level of glutathione that cannot initiate the release of Fenton-catalysed metal ions. Furthermore, the unexpected consumption of oxygen during this oxidation also restricted glucose oxidation. These factors significantly impeded ROS generation and consequently diminished the therapeutic efficacy.

#### Starvation/phototherapies

Phototherapies including photothermal therapy (PTT) and photodynamic therapy (PDT) exert light as an energy source to produce active compounds or heat that prompt the cancer cells’ apoptosis [105–108]. These therapies are usually less invasive and more biocompatible than conventional radiation. Combining starvation therapy with phototherapies presents promising approaches to cancer treatment, offering the enhanced therapeutic efficacy through synergistic mechanisms.

*Starvation/photodynamic therapy*: H_2_O_2_ produced from glucose oxidation can be accumulated to increase endogenous H_2_O_2_ concentration which is usually inadequate for satisfactory PDT results and decomposed into O_2_ under the presence of nanocatalysts. This O_2_ augmentation not only inhibit the activation of defence mechanisms in cancer cells via hypoxia alleviation but also accelerate the formation of singlet oxygen (^1^O_2_)—a crucial compound for PDT by supplying oxygen source [109]. While glucose oxidation cuts off the main energy source and makes cancer cell become more vulnerable, its by-product H_2_O_2_ can be positively used to contribute to amelioration of hypoxia and enhancement of PDT therapeutic effect by providing more oxygen. The singlet oxygen generated from PDT induces cell death via apoptosis, necrosis, and autophagy via diverse signalling pathway related to many factors and caspases [110].

*Starvation/photothermal therapy*: Photothermal therapy works by generating heat to induce cell apoptosis. Due to its independence from ROS generation, PTT is not affected by hypoxia. However, the main barrier of PTT is the release of heat shock proteins (HSPs) that allow cancer cells survival by repairing thermal-induced damage. The combination with starvation, especially glucose oxidation, provides many benefits: (1) the glucose starvation could impede the HSPs expression by blocking the main energy supply used for HSP production [111]; (2) the glucose oxidation consumes oxygen and cause hypoxia; however, PTT is not effected by hypoxia and maintain its therapeutic performance; (3) the mild temperature increase induced by PTT could enhance GOx enzyme efficiency instead of causing adverse effects on its stability [112]. These factors lead to a synergistic enhancement of therapeutic outcomes when photothermal and starvation therapies are combined. Table 4 summarises recent nanomaterials-mediated starvation/phototherapies.

**Table 4:** Representative recent nanomaterials-mediated starvation/phototherapies

Materials	Starvation causing agent	PTT agents	PDT agents	Cell lines	Times of *in viv*o treatment (days)	Tumour suppression (S) or eradication (E)	Ref
f UCNPs@mSiO_2_@CeO_2_-GOD	GOx		CeO_2_	4 T1	14	S	[109]
HMnO_2_ nanospheres carried Ce6, GOx	GOx		MnO_2_/Ce6	4 T1 A549	14 (A549 tumours)	S	[113]
Glucose transporter 1 inhibitor genistein (Gen) and Ce6 NPs	Gen		Ce6	LLC	14	E	[114]
YOF:Nd^3+^@MnO_2_ − ICG-GOx-LF	GOx		MnO_2_/ICG	L929	9	S	[115]
Aggregation-induced emission luminogens (AIEgens) and proton pump inhibitors (PPI)	PPI		AIEgens	MGC803	16	S	[116]
Meso porous silica (mSiO_2_)-shell-wrapped NaErF_4_@NaYF_4_ nanoparticles (LnNP@mSiO_2_)–loaded Ce6 and 2-DG	2-DG		Ce6 (Er^3+^ support)	HCT116	14	E	[117]
H-MnO_2_/Ce6/GOx/F-127	GOx		MnO_2_/Ce6	EMT-6	14	S	[118]
GOx-MSN@MnPc-LP	GOx		MnPc	4 T1 HeLa	14	E	[119]
Dual-locked porphyrin/enzyme-loading ZIF nanoplatform	GOx		Catalase/porphyrin	4 T1	14	E	[120]
Liposome-loaded chlorine e6 (Ce6) and 3-bromopyruvate (3BP)	3BP		Ce6	HeLa	16	S	[121]
Enzyme nanogel (rGCP nanogel) loade porphyrin and GOx	GOx		Catalase/porphyrin	HeLa MCF7 4 T1	13 (4 T1 tumours)	S	[122]
Liquid metal nanoparticles (gallium indium)@GOx	GOx	Liquid metal NPs		4 T1	16	E	[112]
Ag_2_S@mesoporous silica nanoparticles–loaded tirapazamine and GOx	GOx	Ag_2_S		HeLa	14 (U14 tumours)	S	[123]
Narrow-bandgap conjugated polymer (DPQ)–loaded 2-DG	2-DG	DPQ		4 T1 NIH-3 T3	15	S	[124]
Covalent organic framework (COF)–based GOx	GOx	COF		HeLa	14	S	[125]
GOx and Ag NPs functionalised MOFs	GOx	Ag		HeLa	14	S	[126]
Hollow mesoporous silica–loaded 3,3′,5,5′-tetramethylbenzidine (TMB) and GOx	GOx	TMB		4 T1	16	E	[127]
Nanoplatform UM@ICG@GOX@HA (UiO66, indocyanine green (ICG), MnO_2_, HA)	GOx	ICG (MnO_2_ support)		CT26	14	S	[128]
Nanozyme-laden intelligent macrophage express based on IR820-macrophage loaded with GOx	GOx	IR820		4 T1	20	S	[129]
Heptamethine cyanine (Cy7)–GOx	GOx	Cy7		4 T1	21	S	[130]
TiO_2_-x@POMs-GOD	GOx	TiO_2-x_ quantum dots		MEF	No *in vivo*		[131]
Glucose oxidase (GOX), indocyanine green (IR820), and α-cyano-4-hydroxycinnamic acid (CHC) NPs	GOx (CHC support)	IR820		HCT116 CT26	15 (CT26 tumours)	S	[132]
AuNRs@MnO_2_@SiO_2_-loaded GOx	GOx (MnO_2_ support)	AuNRs		4 T1 HEK 293	20 (4 T1 tumours)	S	[133]
Pt-decorated hollow Ag − Au trimetallic nanocages–loaded GOx	GOx	Pt-decorated hollow Ag − Au		4 T1	20	S	[134]
Prussian blue (PB)–loaded hexokinase	Hexokinase	PB		4 T1	14	E	[135]
Conjugated polymer nanoparticles (CPNs-G)–loaded GOx	GOx	Poly-5,5′-(2,5-bis(2-octyldo-decyl) 3,6-di(thiophen-2-yl)−2,5-dihydropyrrolo [3,4-c] pyrrole-1,4-dione		MCF7	No *in vivo*		[136]
ZIF@GOx@AuNRs@eM	GOx	AuNRs		HCT116	14	S	[137]

Organic photosensitisers, including Chlorin e6 and porphyrin, are the most ubiquitous components used in PDT nanotherapeutics. They have minimal long-term side effects and are less invasive compared to drugs used in conventional therapies. Moreover, PDT exhibited highly accurate targeting of tumour tissue due to the dual selectivity on localisation of photosensitiser and confinement of light. Manganese and its derivatives such as oxides played a pivotal role in supporting the PDT process because of their catalytic activity in the decomposition of H_2_O_2_ into O_2_ (catalase mimicking). Various nanoparticles—ranging from organic dyes (e.g. IR780, IR820) to plasmonic metal NPs (e.g. Ag, Au) and 2D materials (e.g. quantum dot, graphene)—were explored as photothermal agents in PTT. Starvation-primed phototherapies often utilised GOx as a starvation-inducing agent. GOx not only catalysed the oxidation of glucose to generate H₂O₂, which is vital for enhancing PDT, but its activity is also amplified under increased temperatures. Conversely, glycolysis inhibitors, such as hexokinase, 2-DG, and 3-BP, were less commonly used as they primarily interrupt glycolysis and not be influenced by temperature changes or enable the generation of H_2_O_2_—crucial factors for synergetic enhancement.

**Fig. 4:**
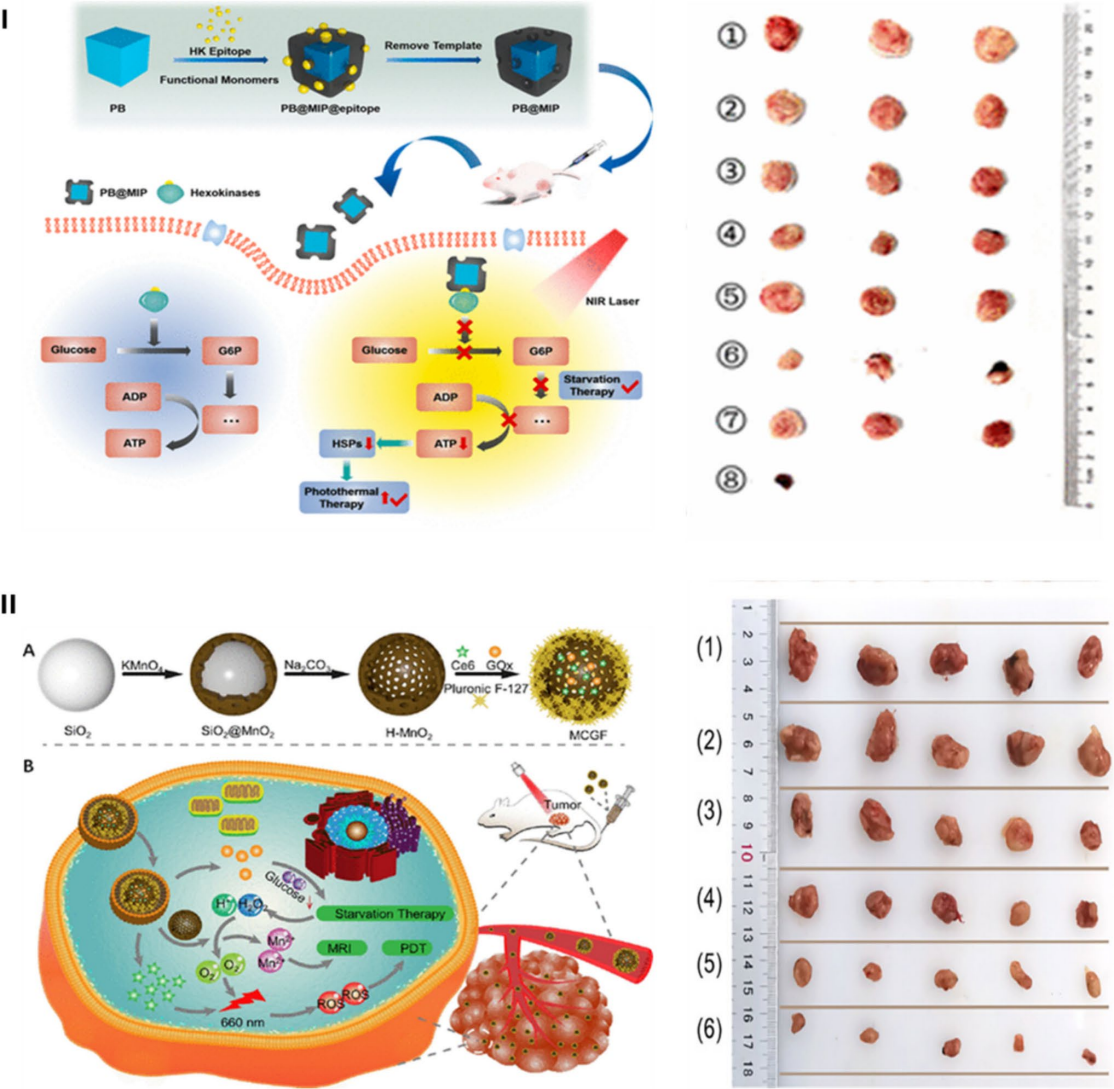
Nanomaterial mediated the synergic starvation/phototherapies. **I** Schematic diagram of the preparation of PB@MIP and its application as a hexokinases inhibitor for combined starvation and enhanced photothermal therapy of malignant tumours (left) and relative tumours’ photographs of different groups: (1) PBS, (2) PBS + NIR, (3) PB, (4) PB + NIR, (5) PB@NIP, (6) PB@NIP + NIR, (7) PB@MIP, (8) PB@MIP + NIR after 14 days of treatment (right). **II** (A) Schematic design of the MCGF nanoplatform and (B) application of MCGF in tumour therapy and imaging, including MRI and enhanced starvation/PDT (left). Photographs of tumours collected from sacrificed mice of different groups (1) PBS, (2) PBS + L660, (3) Ce6 + L660, (4) MCGF, (5) MCF + L660, (6) MCGF + L660 (right). Reproduced with permission. Copyright 2023. American Chemical Society[118] [135]

Certainly, these combinatory therapeutic modalities offer many advantages over the conventional single starvation treatment, but there are many notable challenges to overcome. The phototherapies occur only at irradiated sites selectively; therefore, it is difficult to eradicate metastatic and deeply embedded tumours where the light penetration is attenuated by tissues. This limitation can lead to tumour relapse. Additionally, increasing the temperature during PTT can potentially damage surrounding healthy tissues and induce the production of heat shock proteins that confer heat resistance in cancer cells [138,139]. Figure 4 exhibits 2 nanomaterials that mediated the combination of starvation and phototherapies.

#### Starvation/chemotherapy

During glucose oxidation, glucose oxidase consumes intracellular oxygen to convert glucose into gluconic acid and hydrogen peroxide (H₂O₂), resulting in a localised increase in intracellular acidity and the induction of hypoxic conditions. This alteration of the tumour microenvironment can be strategically exploited to activate and release anticancer drugs and prodrugs. This not only allows localised therapeutic effects but also mitigates side effects via preventing the premature leakage of hydrophilic chemotherapeutic agents during systemic transportation. Furthermore, the starvation-induced vulnerability of tumour cells limits their ability to develop resistance and make them more susceptible to chemotherapy. Co-loading starvation causing agents and anticancer drugs into nanocarriers offers several advantages for achieving synergistic therapy as well as overcoming barriers via highly precise-targeting delivery and enhanced permeability and retention (EPR) effect [140] [141] (Table 5).

**Table 5:** Representative recent nanomaterials-mediated starvation/chemotherapy

Materials	Starvation causing agents	Chemotherapy agents	Targeting agents	Cell line	Times of *in vivo* treatment (days)	Tumour suppression (S) or eradication (E)	Ref
GOx- and TPZ-co-loaded oDex-SeSe-Gel	GOx	Tirapazamine (TPZ)		B16-F10	20	S	[140]
PTX-ASC-GO@MPCSApt NPs	GOx	PTX	Aptamer	4 T1	15	S	[141]
Sorafenib (Sor) and GOx co-loaded into a N-acetyl-galactosamine (GalNAc) modified (ZIF-8)	GOx	Sor	GalNAc	C5 WN1	14	S	[142]
Liposomes co-delivered cisplatin (CDDP) and bis-2-(5-phenylacetamido-1,3,4-thiadiazol-2-yl)-ethyl-sulfide (BPTES)	BPTES	CDDP	Liposomes	SKOV3DDP	16	E	[143]
Banoxantrone (AQ4 N)/GOx@ZIF-8@Cell-membrane(CM)	GOx	AQ4 N	CM	HepG2	21	S	[144]
Hollow mesoporous organosilica-GOx/DOX-CM	GOx	DOX	CM	HepG2	12	S	[145]
Yolk − shell-mesoporous-organosilica-GOx/DOX@aptamer	GOx DOX	DOX	aptamer	MCF-7	21	S	[146]
Folic acid (FA)-functionalised-carbon-dots (CDs)-embedded -with-GOx-and-paclitaxel (PTX)	GOx	PTX	FA	MDA-MB-468	No *in vivo*		[147]
GOx with Pt-NPs in a PLGA -coated-nano-system	GOx	Pt	PLGA	CT26	20	S	[148]
Mesoporous-silicon(DMSN)-binding-peptide, (FTH1)-co-loaded-TPZ-and-GOx	GOx	TPZ	Peptide	L-02 A549	14	S	[149]
GOx-coupled-Ag@mSiO_2_-TPZ	GOx	TPZ		MCF7	No *in vivo*		[150]

Despite notable improvement in tumour suppression efficacy, this combinatory therapeutic approach inherits many challenges of conventional chemotherapy, including drug resistance in cancer cells and toxicity associated with chemodrugs. Furthermore, the combination strategy primarily relies on the tumour microenvironment (TME) for drug release rather than addressing or mitigating its adverse effects. Consequently, the accumulation of H_2_O_2_ and hypoxia leads to the activation of hypoxia-induced factors (HIF) that boost the defence mechanism of cancer cells against therapy.

#### Starvation/gas therapy

The starvation/gas therapy modality leverages H₂O₂ generated during glucose oxidation to oxidise L-arginine (L-Arg), an endogenous nitric oxide (NO) donor, into L-citrulline, thereby releasing NO gas. Compared with using exogenous NO donors, this process overcomes the low-specific delivery to tumours and improves gas therapy notably as well. However, supplementation of free L-arginine can cause a serious increase in plasma L-arginine concentration. High levels of L-arginine could be toxic and cause unpleasant side effects including high potassium levels, nausea, and diarrhoea. Nano-sized delivery vehicles provide the ideal solution to this problem. Covalent organic frameworks (COFs) [151], MnO_2_-HSA-FA [152], MnO_2_-modified poly-dopamine (PDA) and FA [153], FA-BSA/GOx@ZIF-8-L-Arg [154], and tetrasulfide bond–doped mesoporous silica nanoparticles co-loaded GOx and L-Arg [155] contributed promising results in eliminating HeLa cell tumours via synergistic starvation-NO gas therapy. The modification with folic acid (FA), human serum albumin (HSA), and bovine serum albumin-FA (BSA-FA) promoted the targeting delivery via specific recognition of tumours.

In this therapeutic approach, starvation was often induced by glucose oxidation, which generated key factor H_2_O_2_ to support the cooperative dual therapy. Therefore, this approach inherited almost limitations of both glucose oxidation and gas therapy. The combination of gas therapy and other starvation strategies is limited. Moreover, during systemic transportation, GOx could consume blood glucose for oxidation and release H_2_O_2_ that not only causes oxidative stress on normal tissues and diminishes blood glucose concentration but also interacts with L-arginine (both in blood and nanocarriers) leading to uncontrolled release of toxic gas NO. These are potential safety risks which should be considered carefully.

#### Starvation/sonodynamic therapy

Ultrasonic-triggered sonodynamic therapy (SDT) is a novel and promising approach in cancer treatment in recent years, offering advantages in deep-tissue penetration compared to light-based therapies such as PTT or PDT [156]. SDT involves the employment of nanoparticles when exposed to ultrasound, undergoing a process called sonoluminescence, leading to the generation of ROS within the tumour tissue. These ROS induce oxidative stress and cause damage to cancer cells, ultimately leading to cell death. SDT shares the same mechanism as PDT, with the only difference being the replacement of light with ultrasound irradiation. Therefore, glucose oxidation can enhance SDT through the same mechanism underlying combined starvation and photodynamic therapies [157] (Table 6).

**Table 6:** Representative nanomaterials-mediated starvation/sonodynamic therapy

Materials	Starvation causing agent	SDT agents	O_2_ generating agents	Cell lines	Times of *in vivo* treatment (days)	Tumour suppression (S) or eradication (E)	Ref
TiO_2_@Pt/GOx	GOx	TiO_2_@Pt	Pt	4 T1	14	E	[157]
Hollow CoP@N − carbon@PEG	CoP@N − carbon			4 T1 L929	14 (4 T1 tumours)	E	[158]
Porphyrin-based-PCN-224-loaded-Pt and GOx	GOx	Tetrakis(4-carboxyphenyl)porphyrin	Pt	BxPC-3	15	S	[159]
AuPt@MgSiO_3_@GOx	GOx	AuPt@MgSiO_3_		MCF-7	13	S	[160]
Organoplatinum (II) complex (Pt-TPE)	Pt-TPE			4 T1	14	S	[161]

Sonodynamic therapy has emerged as a non-invasive therapeutic strategy which has fewer side effects and is a better choice for deep tumours due to ultrasound penetration ability. However, research in this novel area is quite limited. Moreover, the combination with starvation treatment is nearly based on the application of glucose oxidase. Further exploration in this area could focus on optimising the combination of SDT with various starvation strategies to yield significant advancements in cancer treatment, especially for hard-to-reach tumours.

#### Other starvation-based dual therapies

Besides these integrated therapies above, some new approaches have been reported. A multistage responsive dual-enzyme nano-cascade was applied for starvation-enhanced radiotherapy via glucose depletion facilitating the faster-kill radiotherapy. In this study, two enzymes GOx and CAT were placed closely within a polymeric coating for the continuous multistage process to prevent the escape of H_2_O_2_ causing oxidative stress [162]. Binding siRNA and GOx on Au NRs also gives a more promising combined therapy in which GOx deprives glucose and siRNA has significant effects on cancer growth, metastasis, and drug resistance [163]. Porous Pt binding with GOx provided new starvation/electrodynamic therapy. Pt not only replenished O_2_ via catalysing H_2_O_2_ decomposition but also generated ROS under an alternating electric field [164]. For integrated starvation/immunotherapy, PCP-Mn-DTA@GOx@1-MT [165] and microalgae-integrated living hydrogel [166] have been administered.

#### Multifunctional nanomaterial–crucial key in starvation-based multitherapy

The explosion of research focusing on multifunctional nanomaterials for multimodal therapies (> 3 modalities) in cancer treatment (Table 7) over the past few years reflects the growing recognition of their potential to overcome the limitations of monotherapy and to enhance therapeutic efficacy synergistically. This literature review will update elaborately on recent efforts in the last 3 years.

**Table 7:** Representative nanomaterials-mediated starvation-based multimodal therapies

Nanomaterials	Multi-therapeutic combination	Starvation causing agents	Roles of active agents in the nanomaterials	Cell line	Duration of *in vivo* treatment (days)	Tumour suppression (S) or eradication (E)	Ref
**3 therapies combination**							
Dendritic-mesoporous-coppery-carbon nanosphere (Cu-MCGH)@GOx	ST/CDT/PTT	GOx	Cu-MCGH-CDT, PTT	4 T1	14	S	[111]
Ruthenium-nanoaggregate (RuNA) @MnO_2_@GOx			MnO_2_-CDT, RuNA-PTT	4 T1	13	E	[167]
Cu-doped-mesoporous-Prussian-blue (PB)@GOx			PB-PTT, Cu-CDT	4 T1	14	S	[168]
Hollow-porous-carbon-coated-FeS_2_(HPFeS_2_@C)@GOx			FeS_2_-PTT, CDT	HeLa	14	S	[169]
MoO_3−x_@Fe_3_O_4_‑GOx-PVP			Fe_3_O_4_-CDT, MoO_3−x_-PTT	A549	16	S	[170]
GOx@CuS			CuS-PTT, CDT	B16 F10	9	S	[171]
CuS@GOx/atovaquone(ATO)			CuS-CDT, PTT	FLS	45	S	[172]
Bi/Cu-gallic acid (GA) encapsulated GOx			Cu-CDT, Bi-PTT	4 T1	18	S	[173]
Cobalt-based ZIF67-ICG/tamoxifen (TAM)@GOx			ICG-PTT, cobalt-based ZIF67-CDT	MCF-7	15	S	[174]
MoS_2_-ALG-Fe/GOx			Fe-CDT, MoS_2_-PTT	HT29	14	S	[175]
CoMnFe-layered double oxides@GOx			CoMnFe-CDT, PTT	4 T1	11	S	[176]
CuS@Axitinib-SiO_2_@2-DG-CaCO_3_-RGD		2-D, G, CaCO_3,_ Axitinib	CuS-PTT, CDT	4 T1	15	S	[177]
AuPtAg-GOx	ST/PTT/IT	GOx	AuPtAg-O_2_ replenishing, PTT	4 T1	14	S	[178]
ZIFs-derived-CuCo(O)/GOx@PCNs			CuCo(O)-O_2_ replenishing, PTT, IT	4 T1	9	S	[179]
Nanoliposome-loaded GOx and TMB			TMB-PTT	4 T1	14	E	[180]
DMSN@Au@ immunostimulatory(R837)		Au	Au-PTT, R837-IT	4 T1	30	S	[181]
G5-PEG-LyP-1-CuS-DMXAA		DMXAA	CuS-PTT	4 T1	18	S	[182]
PtPd@GOx@IR780	ST/SDT/PTT	GOx	PdPt-PTT, O_2_ replenishing, IR780-SDT	4 T1	14	S	[183]
B16 F10—graphene oxide (GO)—Heparin-3-bromopyruvate (3BP)–loaded etoposide (EPT)	ST/CT/PTT	3BP	GO-PTT, EPT-CT	B16 F10	14	S	[184]
MoS_2_@DOX/GOx@MnO_2_	ST/PTT/CT	GOx (MnO_2_ support)	DOX-CT; MoS_2_-PTT	HepG2	14	S	[185]
Phenylboronic-acid-modified-donor–acceptor–donor molecule (BTP)/DOX/2DG		2-D,G	BTP-PTT, DOX-CT	143B	12	S	[186]
Mesoporous-silica-nanorods@GOx@DOX@PDA		GOx	DOX-CT, PDA-PTT	HepG2 HL7702	No *in vivo*		[187]
(GOx)-attached Fe_3_O_4_-loaded pro-DOX	ST/CDT/CT	GOx	Fe_3_O_4_-CDT, pro-DOX-CT	MCF-7 MCF-7/Adr	20	S	[188]
HSA–GOx–TPZ–Fe^3+^–TA			TPZ-CT, Fe^3+^-CDT	4 T1	14	S	[189]
Mil101(Fe)@GOx/DOX@FA-TPP			DOX-CT, Fe-CDT	4 T1	14	S	[190]
MnFe-based MOFs@Au@ cisplatin-prodrug (DSCP)		Au	MnFe-based MOFs-CDT, DSCP-CT	B16 F10	21	S	[191]
Mn_3_O_4_ decorated dendritic mesoporous organosilica@GOx@ IDO inhibitor Epacadostat (IDOi)	ST/CDT/IT	GOx	IDOi-IT, Mn_3_O_4_-CDT	4 T1	14	S	[192]
MnO_2_@ Methoxy-poly(ethylene–glycol) (mPEG)-phenylboronic acid-modified-generation5(G5-mPEG − PBA)@GOx@ cyclic-GMP-AMP(cGAMP)			MnO_2_-CDT, cGAMP-IT	CT26	21	E	[193]
Ag@PDA/GOx/TPZ@M	ST/CT/metal ion	GOx	TPZ-CT, Ag@PDA-metal ions therapy	HeLa	13	E	[194]
Bi/BiVO_4_-loaded GOx and diallyl trisulfide (DATS)	ST/GT/SDT	GOx	Bi/BiVO_4_-SDT, DATS-GT	4 T1 L929	14	E	[195]
PtMo-Au	ST/SDT/CDT	Au	PtMo-Au-SDT, CDT	4 T1	18	E	[196]
Porphyrin-containing-covalent-organic-polymer (PCOP)@GOx	ST/PDT/CDT	GOx	Fe-CDT, porphyrin-PDT	HeLa MCF-7 L929	18 (MCF-7 tumours)	S	[197]
**4 therapies combination**							
Fe- semiconducting polymer dot modified (Pdot@Fe) with GOx	ST/PTT/PDT/CDT	GOx	Fe-CDT, Pdot-PTT, PDT	MCF-7	14	E	[198]
Ce6-loaded-H-CeO_2_@PDA@GOx			CeO_2_/Ce6-PDT, CDT; PDA-PTT	T98G	No *in vivo*		[199]
Erythrocyte-membrane-encapsulated-GOx-and-manganese/ferrite			Mn/Fe-CDT, PTT, PDT	4 T1	14	S	[200]
ICG/Au/Pt@PDA − PEG		Au	Pt-CDT, Pt-ICG-PDT, PDA-PTT	B16 F1	No *in vivo*		[201]
HM-CuS NPs as Temozolomide (TMZ). GOx, Lactoferrin(Lf)	ST/PTT/CDT/CT	GOx	CuS-PTT, CDT; TMZ-CT	C6	10	S	[202]
Cu_9_S_8_ @ AQ4 N @ GOx			AQ4 N-CT, Cu_9_S_8_-PTT, CDT	GL261	10	S	[203]
Fe_3_O_4_@ZIF-8/GOx@MnO_2_	ST/PTT/CDT/IT	GOx	MnO_2_ -O_2_ replenishing, Fe_3_O_4_-PTT, CDT, trigger IT	4 T1	9	E	[204]
Chitosan(CS) hydrogel co-loaded L-Arg and GOx@Cu/Zn-MOF	ST/PTT/PDT/GT	GOx	L-Arg-GT, Cu/Zn-MOF-PTT, PDT	4 T1	14	S	[205]
Fe/ZIF-8@GOx@L-Arg@adriamycin-hydrochloride (Dox)	ST/CDT/CT/GT	GOx	Fe-CDT, L-Arg-GT, Dox-CT	MCF-7/Adr 4 T1	15 (4 T1 tumours)	E	[206]
TiO_2−x_@Cu,S-MONs@GOx	ST/GT/PTT/CDT	GOx	Cu-CDT, S-GT, TiO_2-x_-PTTMON	4 T1	14	E	[207]

*CDT* chemodynamic therapy, *PTT* photothermal therapy, *PDT* photodynamic therapy, *SDT* sonodynamic therapy, *CT* chemotherapy, *ST* starvation therapy, *IT* immunotherapy, *GT* gas therapy, *GeT* gene therapy*.* Tumour eradication means the disappearance of tumours in some mouse (not all mouse)

Like dual starvation–based therapies, multifaceted therapeutic approaches relied virtually on glucose oxidation to trigger the scarcity of glucose in tumours via glucose oxidase (GOx) and its mimicking nanoparticles (e.g. Au NPs). The utilisation of starvation-inducing agents such as glucose inhibitors or blood occlusion agents is rare. Therefore, the adaptation of cancer cells to glucose deprivation via metabolic changes was still the main barrier. However, the combination of starvation and other therapies could attack the tumours more effectively and eradicate them quickly before the development of therapeutic resistance. Moreover, the coalition of many treatments could take advantage of the limitations of starvation in a more effective way when compared to mono and dual therapies. For example, starvation/chemodynamic (dual combination) will only consume a certain amount of ubiquitous H_2_O_2_ by-product of glucose oxidation to trigger the synergistic treatment. The remaining amount of this compound still exists in the cell environment and continues causing oxidative stress. Therefore, the combination with one more therapeutic strategy that uses H_2_O_2_ to enhance therapeutic effects such as sonodynamic, photodynamic, or gas therapy is considered a useful solution. These approaches not only utilise more H_2_O_2_ to alleviate intra-tumoural-oxidative stress in a better way but also significantly intensify therapeutic outcomes on tumour suppression and eradication.

Starvation-based multimodal therapies practically employ together many functional materials which have different roles in the treatment. Metals and metal compounds (oxides, sulfides, ferrites, MOF, etc.) based on Fe, Mn, Cu, Co, Zn, Ru, Mo, Ce, Bi, etc. were used as key nanoparticles that not only trigger the other therapies (PTT, PDT, CDT, SDT) but also support the starvation. The noble metal nanoparticles such as Pt, Ag, Pd, and, especially, Au could play a dual role, as the starvation-inducing agent via the mimicking of glucose oxidase and as the other therapies’ active agent. Some organic compounds such as IR780, Prussian blue and L-Arginine were also applied as active agents in combined therapies.

As seen in Table 7, some multifunctional materials for multimodal therapies could eradicate the tumours in a short time of treatment (13–21 days). The multifaceted therapeutic approaches could open the promising prospect of completely curing cancer. Eradicating tumours in a short time could not only restrict the adaptation of tumours against the treatment but also improve patient compliance compared to conventional treatments. However, the combination of many materials could increase the toxicity of normal cells and cause more side effects. The highly targeted delivery is still required to not only promote synergistic therapeutic effects but also prevent adverse effects on normal tissues.

**Fig. 5:**
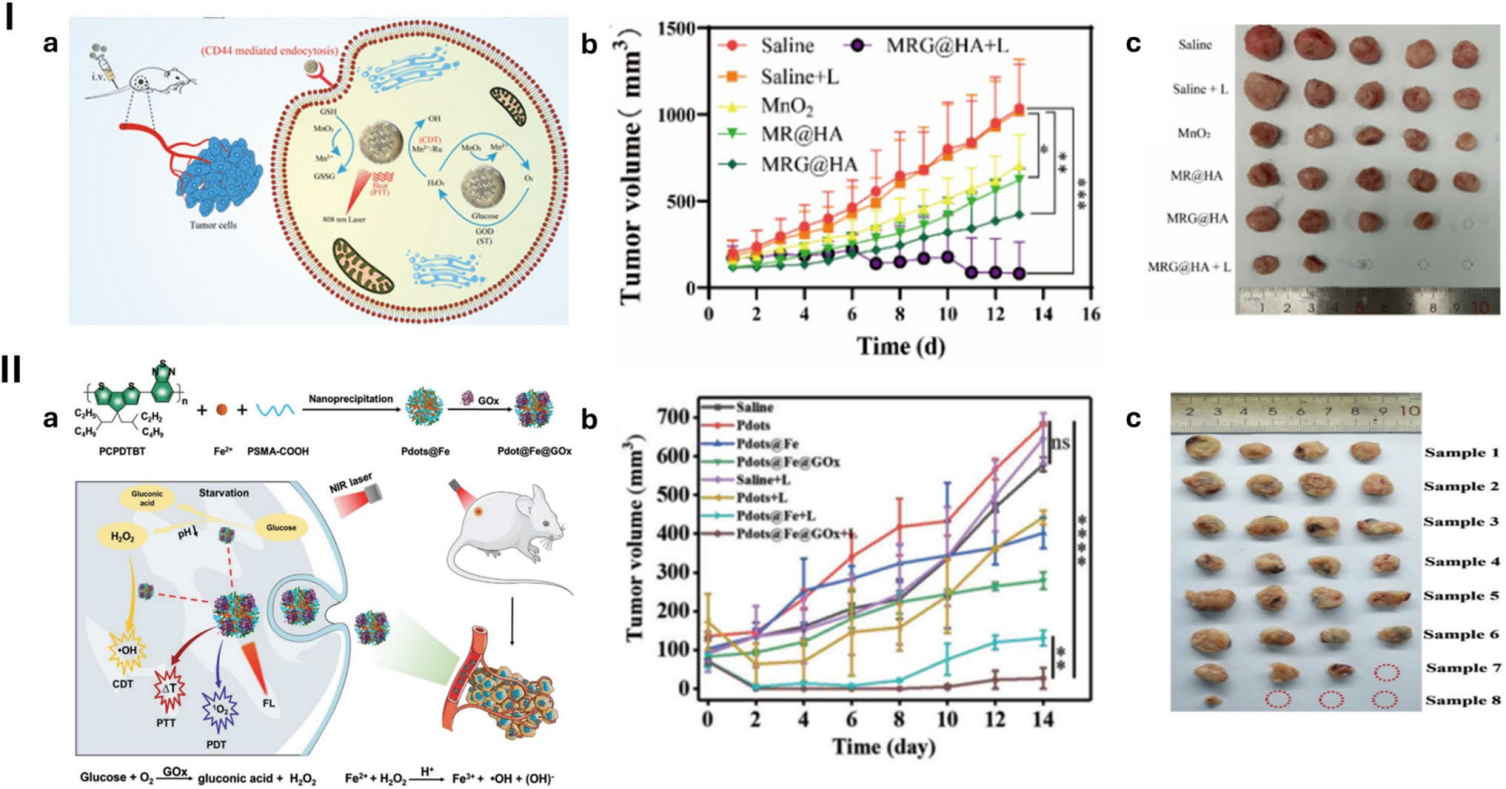
Nanomaterial mediated starvation-based multimodal therapies. **I** (**a**) Schematic diagram of preparation and mechanism of ruthenium-nanoaggregate (RuNA)@MnO2@GOx (MRG@HA). (**b**) Tumour volume change curves of mice during treatment. (**c**) Tumour shape and size of mice at the end of treatment. Copyright 2023 American Chemical Society [167]. **II** (a) Schematic illustration of the preparation of Pdot@Fe@GOx for enhanced multimodal cancer treatment. (b) Time-dependent tumour growth curves. (c) Digital photographs of the dissected tumours of different groups Saline, P dots, P dots@Fe, P dots@Fe@GOx, Saline + L (light), P dots + L, P dots@Fe + L, P dots@Fe@GOx + L. Copyright 2023 Wiley Periodicals LLC [198]. Reproduced with permission

Furthermore, metabolic-intervened starvation therapies usually focused on single nutrient blocking. This cannot cause the severe famines to completely dislodge the tumour and usually facilitates tumour metabolic adaptations to treatment. The multi-nutrient-based starvation should be considered to improve therapeutic effectiveness. Facing the depletion of multiple nutrients, tumour starvation will be more stringent and induce cell death more easily. Tumours need more time and effort to develop metabolic change to promote survival. The concomitant blocking of many nutrients could be an effective way to prevent the emergence of therapeutic-resistant adaptation in cancer cells. However, multi-nutrient deprivation can worsen adverse effects on normal tissues. Figure 5 exhibits 2 strategies that were applied for multitherapy modalities based on starvation.

## Advantages and limitations

### Advantages

Advancements in knowledge about not only cancer cells but also multifunctional nanomaterials opened doors to diagnose and treat cancer in more effective ways. The barriers of monotherapy based on starvation can be overcome via the combination of therapies. Starvation therapy usually enervates cancer cells and delays their growth instead of completely eradicating them. Tumours become more vulnerable after starvation. Hence, the efficiency of other therapies can be improved significantly in starvation-based multi-therapeutic modalities. This facilitates the abridgement of treatment duration. According to *in vivo* anti-tumour studies of starvation-based multitherapy, optimistic results in tumour demolition can be obtained in short periods from 9 to 20 days. This will be extremely beneficial for patients in clinical trials because long treatment periods of conventional therapies can cause exhaustion.

The promising effects on abolishing the cancer progression were attained in various cell lines, such as HeLa, 4 T1, MCF-7, and HepG2. Therefore, the integration of starvation and other therapies can provide effective treatment for various cancer types instead of just a specific one. Cancer cells often develop resistance to single-agent therapies through various mechanisms, including metabolic changes and activation of survival pathways. Multi-therapeutic modalities that target multiple pathways simultaneously can help overcome treatment resistance and prevent the rapid adaptation of cancer cells to therapy.

Nanocarriers can be designed to selectively deliver both starvation causing agents and anticancer drugs to tumour tissue, minimising off-target effects on healthy tissues. By incorporating targeting ligands or responsive elements, nanocarriers can further enhance their specificity for cancer cells, ensuring precise delivery of therapeutic agents to the desired site but restricting the adverse effects on surrounding tissues as well. Therefore, starvation-based multitherapy is less invasive and more compatible. It can be a desirable alternative to conventional ones.

### Limitations and clinical challenges

While there have been promising outcomes in preclinical studies of starvation-based synergistic therapy, clinical trials involving nanomaterials are still limited.

Firstly, the adverse effects caused by the accumulation of nano-sized particles should be considered carefully, especially nanomaterials based on non-dietary elements. Most studies used cytotoxicity test to conclude biocompatibility of nanomedicines; however, their long-term effects and degradation are also the matters of concern. The life of mice used for *in vivo* experiment is too short for biosafety evaluation. In addition, some nanomaterials based on human essential minerals such as iron, copper, or manganese are considered less harmful than non-dietary elements; however, their degradation may cause side effects related to the increase of their concentration in body that could lead to serious damages to organs. The promising preclinical cytotoxicity test cannot ensure absolute safety when administering nanoparticles in humans. Therefore, the degradation pathways of nanomedicines after exerting their therapeutic effects represent a crucial gap that needs to be explored for future clinical translation. Moreover, multifunctional nanoparticles can result in more serious metabolic risks in normal cells if they are leaked during the transportation inside the body. The highly targeting delivery to confine unwanted side effects is also necessary.

Secondly, some novel approaches to starvation therapy are still in the early stages of development and require thorough investigation—for example, lactate deprivation, amino acid depletion, and the combination of sonodynamic and starvation therapies. Reports in these areas are still limited.

Thirdly, most combinations of starvation and other therapies rely on glucose oxidation. However, not all cancer cells rely on same metabolic pathways. Some cancer cells are vulnerable to the scarcity of glucose while others seem to be more resistant and continue to survive in low-glucose environments via gene alterations and metabolic adaptions that allow them to consume the other energy sources such as amino acid and lactate. Hence, there is still ample room to explore various approaches for combining starvation therapy with other treatments. However, the other strategies of starvation such as amino acid depletion or lactate deprivation do not produce by-product that allow synergistic therapeutic performance like H_2_O_2_.

Fourthly, the scaling up of nanomedicines also presents significant challenges. The synthesis and fabrication of these active compounds usually require complicated processes with intricate equipment. The minor errors in scaling up process can result in the change in structure and physicochemical properties of nanomedicines that compromise their therapeutic performance. For example, gold nanoparticles can effectively mimic glucose oxidase only at small sizes, whereas larger nanoparticles exhibit reduced catalytic activity [208]. Therefore, if scaling up is not carefully conducted to maintain consistency with laboratory conditions, changes in properties such as size and structure of the nanomedicines may occur, potentially affecting therapeutic outcomes. Due to the challenges in scaling up, only small amounts of nanomedicines are produced per synthesis, particularly in low-yield processes. This leads to high costs, making them less affordable for patients. In addition, the difficulty in scaling up is also a major challenge for clinical translation. Clinical trials necessitate the production of substantial quantities of nanoparticles to accommodate investigations involving a large cohort of patients. Laboratory-scale synthesis is typically inadequate to meet this demand.

Fifthly, during the transportation to tumour site, starvation-triggered nanoparticles could interact with immune system and trigger immune-responsive via release of pro-inflammatory cytokine that leads to unintended inflammation and cytokine release syndrome. These could cause severe effects on patient health. In addition, the immune system could trigger the clearance of nanoparticles via activation of mononuclear phagocyte system that diminishes bioavailability and therapeutic outcomes of nanomedicines [209].

Ultimately, the clinical translation of starvation-based therapeutic modalities remains significantly hindered by numerous challenges. These include concerns about biosafety, long-term toxicity, adverse effects on normal cells, degradation pathways, scalability, unintended immunogenicity, and reliance on glucose oxidation, which can lead to metabolic adaptation. Moreover, starvation therapy and its related therapeutic modalities are a broad and complex landscape, encompassing diverse approaches with distinct therapeutic mechanisms. Evaluating the therapeutic performance of a large number of nanomedicines to identify the most promising candidates for clinical trials is challenging due to inconsistencies in treatment conditions, including variations in dosage, mechanisms of action, and treatment duration.

## Conclusion and outlook

Starvation therapy has many approaches including vascular disruption, anti-angiogenesis, metabolic interferences via glucose deprivation, amino acid depletion, and lactate deprivation. Instead of being utilised as a monotherapy, starvation therapy can be combined with other therapies to enhance the therapeutic efficiency synergistically and overcome the barriers related to hypoxic conditions, an increase of intracellular pH and cytotoxicity of by-products. In starvation-based multimodal therapies, not only cellular hypoxia but also intracellular pH can be alleviated considerably via the decomposition of by-products H_2_O_2_ into useful O_2_. Moreover, hypoxic conditions and pH increase can also facilitate the release and activation of anticancer drugs. The targeted delivery of starvation causing agents is improved significantly by using nano-sized carriers. The utilisation of new medicines that support starvation via the blocking of signalling pathways and molecules’ transportation promotes therapeutic efficacy notably. In addition, the starvation caused by blocking the bloodstream is not limited to using conventional medicines.

The administration of nanomedicines provides promising results. Most innovations in this field are still in their infancy, with underlying challenges regarding clinical translation that need to be assessed in detail. There are many challenges to overcome including the safety of treatments using nanomedicines and their adverse effects on normal tissues. The accumulation of nanoparticles that will provide promising tumouricidal effect or cause aberrant metabolism in normal tissues should be considered carefully and meticulously. While limitations and challenges exist, the future of cancer starvation therapy remains promising because of the efficacy it provides. Overall, combining starvation therapy with other treatment modalities offers a promising approach to cancer treatment, capitalising on the metabolic vulnerabilities of cancer cells and enhancing overall treatment efficacy. Continued research efforts are focused on optimising multi-therapeutic modalities and translating them into clinical practice for the benefit of cancer patients.

## Data Availability

No datasets were generated or analysed during the current study.

## References

[1] Anand, U., Dey, A., Chandel, A. K. S., Sanyal, R., Mishra, A., Pandey, D. K., et al. (2023). Cancer chemotherapy and beyond: Current status, drug candidates, associated risks and progress in targeted therapeutics. *Genes & Diseases,**10*(4), 1367–1401. 10.1016/j.gendis.2022.02.007 37397557 10.1016/j.gendis.2022.02.007 PMC10310991

[2] Tang, J. L., Moonshi, S. S., Wu, Y., Cowin, G., Vazquez-Prada, K. X., Tran, H. D., et al. (2025). A methotrexate labelled dual metal oxide nanocomposite for long-lasting anti-cancer theranostics. *Materials Today Bio,**30*, 101377. 39742148 10.1016/j.mtbio.2024.101377 PMC11683249

[3] Ta, H. T., Dass, C. R., Larson, I., Choong, P. F., & Dunstan, D. E. (2009). A chitosan hydrogel delivery system for osteosarcoma gene therapy with pigment epithelium-derived factor combined with chemotherapy. *Biomaterials,**30*(27), 4815–4823. 19505719 10.1016/j.biomaterials.2009.05.035

[4] Ta, H. T., Dass, C. R., Larson, I., Choong, P. F., & Dunstan, D. E. (2009). A chitosan–dipotassium orthophosphate hydrogel for the delivery of doxorubicin in the treatment of osteosarcoma. *Biomaterials,**30*(21), 3605–3613. 19345993 10.1016/j.biomaterials.2009.03.022

[5] Tang, J. L., Moonshi, S. S., & Ta, H. T. (2023). Nanoceria: An innovative strategy for cancer treatment. *Cellular and Molecular Life Sciences,**80*(2), 46. 36656411 10.1007/s00018-023-04694-y PMC9851121

[6] Baskar, R., Lee, K. A., Yeo, R., & Yeoh, K. W. (2012). Cancer and radiation therapy: Current advances and future directions. *International Journal of Medical Sciences,**9*(3), 193–199. 10.7150/ijms.3635 22408567 10.7150/ijms.3635 PMC3298009

[7] Robatel, S., & Schenk, M. (2022). Current limitations and novel perspectives in pancreatic cancer treatment. *Cancers (Basel), 14*(4). 10.3390/cancers14040985 10.3390/cancers14040985 PMC8870068 35205732

[8] Vazquez-Prada, K. X., Moonshi, S. S., Xu, Z. P., & Ta, H. T. (2023). Photothermal nanomaterials for theranostics of atherosclerosis and thrombosis. *Applied Materials Today,**35*,. 10.1016/j.apmt.2023.101967

[9] Anh Tran, N., Seok Song, M., Kim, G., Binh Nguyen, N., Hoàng Ly, N., Lee, S. Y., et al. (2022). Oxygen-replenishing manganese oxide catalytic nanoparticles on removable pipette surfaces for hypoxic tumour photodynamic therapy. *Applied Surface Science,**604*,. 10.1016/j.apsusc.2022.154516

[10] Yang, Z., Yuan, M., Cheng, Z., Liu, B., Ma, Z., Ma, J., et al. (2024). Defect-repaired g-C(3)N(4) nanosheets: Elevating the efficacy of sonodynamic cancer therapy through enhanced charge carrier migration. *Angewandte Chemie (International ed. in English),**63*(18), e202401758. 10.1002/anie.202401758 38320968 10.1002/anie.202401758

[11] Ma, Z., Yuan, M., Cheng, Z., Yang, Z., Yang, L., Liu, B., et al. (2024). A mild and efficient sonothermal tumor therapy enhanced by sonodynamic effect with biodegradable red phosphorus nanoparticles. *Chemical Engineering Journal,**482*,. 10.1016/j.cej.2024.148711

[12] Bai, Y., Liu, M., Wang, X., Liu, K., Liu, X., & Duan, X. (2023). Multifunctional nanoparticles for enhanced chemodynamic/photodynamic therapy through a photothermal, H(2)O(2)-elevation, and GSH-consumption strategy. *ACS Applied Materials & Interfaces,**15*(48), 55379–55391. 10.1021/acsami.3c12479 38058112 10.1021/acsami.3c12479

[13] Goldberg, M. S. (2019). Improving cancer immunotherapy through nanotechnology. *Nature Reviews Cancer,**19*(10), 587–602. 10.1038/s41568-019-0186-9 31492927 10.1038/s41568-019-0186-9

[14] Vargiu, V., Amar, I. D., Rosati, A., Dinoi, G., Turco, L. C., Capozzi, V. A., et al. (2021). Hormone replacement therapy and cervical cancer: A systematic review of the literature. *Climacteric,**24*(2), 120–127. 10.1080/13697137.2020.1826426 33236658 10.1080/13697137.2020.1826426

[15] Yang, B., Ding, L., Chen, Y., & Shi, J. (2020). Augmenting tumor-starvation therapy by cancer cell autophagy inhibition. *Advanced Science (Weinh),**7*(6), 1902847. 10.1002/advs.201902847 10.1002/advs.201902847 PMC7080508 32195096

[16] Liu, Z. L., Chen, H. H., Zheng, L. L., Sun, L. P., & Shi, L. (2023). Angiogenic signaling pathways and anti-angiogenic therapy for cancer. *Signal Transduction and Targeted Therapy,**8*(1), 198. 10.1038/s41392-023-01460-1 37169756 10.1038/s41392-023-01460-1 PMC10175505

[17] Hasani, A., & Leighl, N. (2011). Classification and toxicities of vascular disrupting agents. *Clinical Lung Cancer,**12*(1), 18–25. 10.3816/CLC.2011.n.002 21273175 10.3816/CLC.2011.n.002

[18] Saghafian Larijani, R., Shabani Ravari, N., Goodarzi, N., Akhlaghpour, S., Saghafian Larijani, S., Rouini, M. R., et al. (2022). Current status of transarterial chemoembolization (TACE) agents in hepatocellular carcinoma treatment. *Journal of Drug Delivery Science and Technology,**77*,. 10.1016/j.jddst.2022.103905

[19] Fung, M. K. L., & Chan, G. C. (2017). Drug-induced amino acid deprivation as strategy for cancer therapy. *Journal of Hematology & Oncology,**10*(1), 144. 10.1186/s13045-017-0509-9 28750681 10.1186/s13045-017-0509-9 PMC5530962

[20] Zhang, Y., Li, Q., Huang, Z., Li, B., Nice, E. C., Huang, C., et al. (2022). Targeting glucose metabolism enzymes in cancer treatment: Current and emerging strategies. *Cancers (Basel), 14*(19). 10.3390/cancers14194568. 10.3390/cancers14194568 PMC9559313 36230492

[21] de la Cruz-Lopez, K. G., Castro-Munoz, L. J., Reyes-Hernandez, D. O., Garcia-Carranca, A., & Manzo-Merino, J. (2019). Lactate in the regulation of tumor microenvironment and therapeutic approaches. *Frontiers in Oncology,**9*, 1143. 10.3389/fonc.2019.01143 31737570 10.3389/fonc.2019.01143 PMC6839026

[22] Guelfi, S., Hodivala-Dilke, K., & Bergers, G. (2024). Targeting the tumour vasculature: From vessel destruction to promotion. *Nature Reviews Cancer,**24*(10), 655–675. 10.1038/s41568-024-00736-0 39210063 10.1038/s41568-024-00736-0

[23] Muz, B., de la Puente, P., Azab, F., & Azab, A. K. (2015). The role of hypoxia in cancer progression, angiogenesis, metastasis, and resistance to therapy. *Hypoxia (Auckl),**3*, 83–92. 10.2147/HP.S93413 27774485 10.2147/HP.S93413 PMC5045092

[24] Huang, Y., Gong, P., Liu, M., Peng, J., Zhang, R., Qi, C., et al. (2021). Near-infrared light enhanced starvation therapy to effectively promote cell apoptosis and inhibit migration. *Materials Advances,**2*(12), 3981–3992. 10.1039/d1ma00148e

[25] Mitchell, M. J., Billingsley, M. M., Haley, R. M., Wechsler, M. E., Peppas, N. A., & Langer, R. (2021). Engineering precision nanoparticles for drug delivery. *Nature Reviews Drug Discovery,**20*(2), 101–124. 10.1038/s41573-020-0090-8 33277608 10.1038/s41573-020-0090-8 PMC7717100

[26] Wang, J., Li, Y., & Nie, G. (2021). Multifunctional biomolecule nanostructures for cancer therapy. *Nature Reviews Materials,**6*(9), 766–783. 10.1038/s41578-021-00315-x 34026278 10.1038/s41578-021-00315-x PMC8132739

[27] Agrawal, S., Singh, G. K., & Tiwari, S. (2024). Focused starvation of tumor cells using glucose oxidase: A comprehensive review. *International Journal of Biological Macromolecules,**281*(Pt 3), 136444. 10.1016/j.ijbiomac.2024.136444 39389487 10.1016/j.ijbiomac.2024.136444

[28] Yu, S., Chen, Z., Zeng, X., Chen, X., & Gu, Z. (2019). Advances in nanomedicine for cancer starvation therapy. *Theranostics,**9*(26), 8026–8047. 10.7150/thno.38261 31754379 10.7150/thno.38261 PMC6857045

[29] Gacche, R. N. (2023). Changing landscape of anti-angiogenic therapy: Novel approaches and clinical perspectives. *Biochimica et Biophysica Acta - Reviews on Cancer,**1878*(6), 189020. 10.1016/j.bbcan.2023.189020 37951481 10.1016/j.bbcan.2023.189020

[30] Oguntade, A. S., Al-Amodi, F., Alrumayh, A., Alobaida, M., & Bwalya, M. (2021). Anti-angiogenesis in cancer therapeutics: The magic bullet. *Journal of the Egyptian National Cancer Institute,**33*(1), 15. 10.1186/s43046-021-00072-6 34212275 10.1186/s43046-021-00072-6 PMC13316910

[31] Rajabi, M., & Mousa, S. A. (2017). The role of angiogenesis in cancer treatment. *Biomedicines, 5*(2). 10.3390/biomedicines5020034. 10.3390/biomedicines5020034 PMC5489820 28635679

[32] van Heeckeren, W. J., Bhakta, S., Ortiz, J., Duerk, J., Cooney, M. M., Dowlati, A., et al. (2006). Promise of new vascular-disrupting agents balanced with cardiac toxicity: Is it time for oncologists to get to know their cardiologists? *Journal of Clinical Oncology,**24*(10), 1485–1488. 10.1200/JCO.2005.04.8801 16574996 10.1200/JCO.2005.04.8801

[33] Sidorenko, V., Scodeller, P., Uustare, A., Ogibalov, I., Tasa, A., Tshubrik, O., et al. (2024). Targeting vascular disrupting agent-treated tumor microenvironment with tissue-penetrating nanotherapy. *Science and Reports,**14*(1), 17513. 10.1038/s41598-024-64610-7 10.1038/s41598-024-64610-7 PMC11289491 39080306

[34] Wang, Y. X., De Baere, T., Idee, J. M., & Ballet, S. (2015). Transcatheter embolization therapy in liver cancer: An update of clinical evidences. *Chinese Journal of Cancer Research,**27*(2), 96–121. 10.3978/j.issn.1000-9604.2015.03.03 25937772 10.3978/j.issn.1000-9604.2015.03.03 PMC4409973

[35] Qiu, S., Ge, N. J., Sun, D. K., Zhao, S., Sun, J. F., Guo, Z. B., et al. (2016). Synthesis and characterization of magnetic polyvinyl alcohol (PVA) hydrogel microspheres for the embolization of blood vessel. *IEEE Transactions on Biomedical Engineering,**63*(4), 730–736. 10.1109/TBME.2015.2469735 26302505 10.1109/TBME.2015.2469735

[36] Zhang, C., Ni, D., Liu, Y., Yao, H., Bu, W., & Shi, J. (2017). Magnesium silicide nanoparticles as a deoxygenation agent for cancer starvation therapy. *Nature Nanotechnology,**12*(4), 378–386. 10.1038/nnano.2016.280 28068318 10.1038/nnano.2016.280

[37] Zhu, Y. X., Jia, H. R., Guo, Y., Liu, X., Zhou, N., Liu, P., et al. (2021). Repurposing erythrocytes as a “photoactivatable bomb”: A general strategy for site-specific drug release in blood vessels. *Small (Weinheim an der Bergstrasse, Germany),**17*(34), e2100753. 10.1002/smll.202100753 34259382 10.1002/smll.202100753

[38] Al-Amer, O. M. (2022). The role of thrombin in haemostasis. *Blood Coagulation & Fibrinolysis,**33*(3), 145–148. 10.1097/MBC.0000000000001130 35239615 10.1097/MBC.0000000000001130

[39] Anithkumar, M., Rajan, S., Khan, A., Kaczmarek, B., Michalska-Sionkowska, M., Łukowicz, K., et al. (2023). Glucose oxidase-loaded MnFe2O4 nanoparticles for hyperthermia and cancer starvation therapy. *ACS Applied Nano Materials,**6*(4), 2605–2614. 10.1021/acsanm.2c04960

[40] Fu, L. H., Qi, C., Lin, J., & Huang, P. (2018). Catalytic chemistry of glucose oxidase in cancer diagnosis and treatment. *Chemical Society Reviews,**47*(17), 6454–6472. 10.1039/c7cs00891k 30024579 10.1039/c7cs00891k

[41] Fan, T., Sun, G., Sun, X., Zhao, L., Zhong, R., & Peng, Y. (2019). Tumor energy metabolism and potential of 3-bromopyruvate as an inhibitor of aerobic glycolysis: Implications in tumor treatment. *Cancers (Basel), 11*(3). 10.3390/cancers11030317. 10.3390/cancers11030317 PMC6468516 30845728

[42] Abdel-Wahab, A. F., Mahmoud, W., & Al-Harizy, R. M. (2019). Targeting glucose metabolism to suppress cancer progression: Prospective of anti-glycolytic cancer therapy. *Pharmacological Research,**150*, 104511. 10.1016/j.phrs.2019.104511 31678210 10.1016/j.phrs.2019.104511

[43] Yang, T., Zhang, X., Yang, X., Li, Y., Xiang, J., Xiang, C., et al. (2023). A mitochondria-targeting self-assembled carrier-free lonidamine nanodrug for redox-activated drug release to enhance cancer chemotherapy. *Journal of Materials Chemistry B,**11*(17), 3951–3957. 10.1039/d2tb02728c 37067569 10.1039/d2tb02728c

[44] Butler, M., van der Meer, L. T., & van Leeuwen, F. N. (2021). Amino acid depletion therapies: Starving cancer cells to death. *Trends in Endocrinology and Metabolism,**32*(6), 367–381. 10.1016/j.tem.2021.03.003 33795176 10.1016/j.tem.2021.03.003

[45] Wilder, C. S., Chen, Z., & DiGiovanni, J. (2022). Pharmacologic approaches to amino acid depletion for cancer therapy. *Molecular Carcinogenesis,**61*(2), 127–152. 10.1002/mc.23349 34534385 10.1002/mc.23349

[46] Jimenez-Alonso, J. J., & Lopez-Lazaro, M. (2023). Dietary manipulation of amino acids for cancer therapy. *Nutrients, 15*(13). 10.3390/nu15132879. 10.3390/nu15132879 PMC10346484 37447206

[47] Jiang, J., Batra, S., & Zhang, J. (2021). Asparagine: A metabolite to be targeted in cancers. *Metabolites, 11*(6). 10.3390/metabo11060402. 10.3390/metabo11060402 PMC8234323 34205460

[48] Scott, L., Lamb, J., Smith, S., & Wheatley, D. N. (2000). Single amino acid (arginine) deprivation: Rapid and selective death of cultured transformed and malignant cells. *British Journal of Cancer,**83*(6), 800–810. 10.1054/bjoc.2000.1353 10952786 10.1054/bjoc.2000.1353 PMC2363527

[49] Li, Z., Wang, Q., Huang, X., Yang, M., Zhou, S., Li, Z., et al. (2023). Lactate in the tumor microenvironment: A rising star for targeted tumor therapy. *Frontiers in Nutrition,**10*, 1113739. 10.3389/fnut.2023.1113739 36875841 10.3389/fnut.2023.1113739 PMC9978120

[50] Perez-Tomas, R., & Perez-Guillen, I. (2020). Lactate in the tumor microenvironment: An essential molecule in cancer progression and treatment. *Cancers (Basel), 12*(11). 10.3390/cancers12113244. 10.3390/cancers12113244 PMC7693872 33153193

[51] Sharma, D., Singh, M., Gupta, R., Kumar, V., Kumar, V., & Rani, R. (2022). Intervention on lactate in cancer: A promising approach for the development of cancer therapeutics. *Advances in Cancer Biology - Metastasis,**5*,. 10.1016/j.adcanc.2022.100058

[52] Daverio, Z., Balcerczyk, A., Rautureau, G. J. P., & Panthu, B. (2023). How Warburg-associated lactic acidosis rewires cancer cell energy metabolism to resist glucose deprivation. *Cancers (Basel), 15*(5). 10.3390/cancers15051417 10.3390/cancers15051417 PMC10000466 36900208

[53] Kargozar, S., Baino, F., Hamzehlou, S., Hamblin, M. R., & Mozafari, M. (2020). Nanotechnology for angiogenesis: Opportunities and challenges. *Chemical Society Reviews,**49*(14), 5008–5057. 10.1039/c8cs01021h 32538379 10.1039/c8cs01021h PMC7418030

[54] Zarharan, H., Bagherian, M., Shah Rokhi, A., Ramezani Bajgiran, R., Yousefi, E., Heravian, P., et al. (2023). The anti-angiogenesis and antioxidant activity of chitosan-mediated synthesized selenium-gold nanostructure. *Arabian Journal of Chemistry, 16*(7). 10.1016/j.arabjc.2023.104806

[55] Al-Zabin, A., Abu Thiab, T., Zihlif, M., Al-Hunaiti, A., Al-Ameer, H. J., Al-Awaida, W., et al. (2024). Anti-angiogenic and cytotoxic evaluation of green-synthesized Fe(2)ZnO(4) nanoparticles against MCF-7 cell line. *Biomedical Reports,**20*(3), 36. 10.3892/br.2024.1724 38343659 10.3892/br.2024.1724 PMC10853757

[56] Cao, S., Zhang, W., Pan, H., Huang, Z., Guo, M., Zhang, L., et al. (2023). Bioactive lipid-nanoparticles with inherent self-therapeutic and anti-angiogenic properties for cancer therapy. *Acta Biomaterialia,**157*, 500–510. 10.1016/j.actbio.2022.12.022 36535568 10.1016/j.actbio.2022.12.022

[57] Liu, F., Peng, B., Li, M., Ma, J., Deng, G., Zhang, S., et al. (2022). Targeted disruption of tumor vasculature via polyphenol nanoparticles to improve brain cancer treatment. *Cell Reports Physical Science, 3*(1). 10.1016/j.xcrp.2021.100691. 10.1016/j.xcrp.2021.100691 PMC8863382 35199059

[58] Hong, S., Zheng, D. W., Zhang, C., Huang, Q. X., Cheng, S. X., & Zhang, X. Z. (2020). Vascular disrupting agent induced aggregation of gold nanoparticles for photothermally enhanced tumor vascular disruption. *Science Advances,**6*(23), eabb0020. 10.1126/sciadv.abb0020 32548273 10.1126/sciadv.abb0020 PMC7274768

[59] Li, H., Zhou, S., Wu, M., Qu, R., Wang, X., Chen, W., et al. (2023). Light-driven self-recruitment of biomimetic semiconducting polymer nanoparticles for precise tumor vascular disruption. *Advanced Materials,**35*(24), e2210920. 10.1002/adma.202210920 36938865 10.1002/adma.202210920

[60] Zhu, J., Cai, H., Xu, C., Wang, W., Song, X., Li, B., et al. (2023). Acidity-responsive nanoreactors destructed “Warburg effect” for toxic-acidosis and starvation synergistic therapy. *Small (Weinheim an der Bergstrasse, Germany),**19*(46), e2304058. 10.1002/smll.202304058 37475522 10.1002/smll.202304058

[61] Yang, N., Cao, C., Xu, C., Su, Y., Wang, W., Song, X., et al. (2024). Hypoxia-responsive nanoparticles for tumor-specific starvation therapy via a two-pronged approach. *Chemical Engineering Journal,**485*,. 10.1016/j.cej.2024.150133

[62] Ravichandran, G., Harijan, D., Ganapathy, N., Prabusankar, G., De, A., & Rengan, A. K. (2023). The multifaceted role of degradable cobalt nanoparticles: Dual-target starvation and intracellular acidification engendering LC3-associated whole-cell autophagy. *ACS Materials Letters,**5*(10), 2726–2738. 10.1021/acsmaterialslett.3c00616

[63] Kaya-Tilki, E., Ozturk, A. A., Engur-Ozturk, S., & Dikmen, M. (2024). Enhanced anti-angiogenic effects of aprepitant-loaded nanoparticles in human umbilical vein endothelial cells. *Science and Reports,**14*(1), 19837. 10.1038/s41598-024-70791-y 10.1038/s41598-024-70791-y PMC11349893 39191829

[64] Xiao, M., Shi, Y., Jiang, S., Cao, M., Chen, W., Xu, Y., et al. (2022). Recent advances of nanomaterial-based anti-angiogenic therapy in tumor vascular normalization and immunotherapy. *Frontiers in Oncology,**12*, 1039378. 10.3389/fonc.2022.1039378 36523993 10.3389/fonc.2022.1039378 PMC9745116

[65] de la Torre, P., Perez-Lorenzo, M. J., Alcazar-Garrido, A., & Flores, A. I. (2020). Cell-based nanoparticles delivery systems for targeted cancer therapy: Lessons from anti-angiogenesis treatments. *Molecules, 25*(3). 10.3390/molecules25030715. 10.3390/molecules25030715 PMC7038177 32046010

[66] Al-Janabi, A. H. A., Hayati Roodbari, N., & Homayouni Tabrizi, M. (2023). Investigating the anticancer and anti-angiogenic effects of graphene oxide nanoparticles containing 6-gingerol modified with chitosan and folate. *Cancer Nanotechnology, 14*(1). 10.1186/s12645-023-00222-6.

[67] Egorova, A., Petrosyan, M., Maretina, M., Bazian, E., Krylova, I., Baranov, V., et al. (2023). iRGD-targeted peptide nanoparticles for anti-angiogenic RNAi-based therapy of endometriosis. *Pharmaceutics, 15*(8). 10.3390/pharmaceutics15082108. 10.3390/pharmaceutics15082108 PMC10459007 37631322

[68] Luo, L.-J., Jian, H.-J., Harroun, S. G., Lai, J.-Y., Unnikrishnan, B., & Huang, C.-C. (2021). Targeting nanocomposites with anti-oxidative/inflammatory/angiogenic activities for synergistically alleviating macular degeneration. *Applied Materials Today,**24*,. 10.1016/j.apmt.2021.101156

[69] Shen, R., Peng, L., Zhou, W., Wang, D., Jiang, Q., Ji, J., et al. (2022). Anti-angiogenic nano-delivery system promotes tumor vascular normalizing and micro-environment reprogramming in solid tumor. *Journal of Controlled Release,**349*, 550–564. 10.1016/j.jconrel.2022.07.015 35841997 10.1016/j.jconrel.2022.07.015

[70] Li, B., Chu, T., Wei, J., Zhang, Y., Qi, F., Lu, Z., et al. (2021). Platelet-membrane-coated nanoparticles enable vascular disrupting agent combining anti-angiogenic drug for improved tumor vessel impairment. *Nano Letters,**21*(6), 2588–2595. 10.1021/acs.nanolett.1c00168 33650872 10.1021/acs.nanolett.1c00168

[71] Wen, H., Fei, Y., Cai, R., Yao, X., Li, Y., Wang, X., et al. (2021). Tumor-activatable biomineralized nanotherapeutics for integrative glucose starvation and sensitized metformin therapy. *Biomaterials,**278*, 121165. 10.1016/j.biomaterials.2021.121165 34649197 10.1016/j.biomaterials.2021.121165

[72] Wei, C., Liu, Y., Zhu, X., Chen, X., Zhou, Y., Yuan, G., et al. (2020). Iridium/ruthenium nanozyme reactors with cascade catalytic ability for synergistic oxidation therapy and starvation therapy in the treatment of breast cancer. *Biomaterials,**238*, 119848. 10.1016/j.biomaterials.2020.119848 32062149 10.1016/j.biomaterials.2020.119848

[73] Ji, P., An, B., Jie, Z., Wang, L., Qiu, S., Ge, C., et al. (2023). Genetically engineered probiotics as catalytic glucose depriver for tumor starvation therapy. *Materials Today Bio,**18*, 100515. 10.1016/j.mtbio.2022.100515 36582449 10.1016/j.mtbio.2022.100515 PMC9792908

[74] Fu, Z., Du, H., Meng, S., Yao, M., Zhao, P., Li, X., et al. (2022). Tumor-targeted dual-starvation therapy based on redox-responsive micelle nanosystem with co-loaded LND and BPTES. *Materials Today Bio,**16*, 100449. 10.1016/j.mtbio.2022.100449 36238964 10.1016/j.mtbio.2022.100449 PMC9552111

[75] Du, H., Meng, S., Geng, M., Zhao, P., Gong, L., Zheng, X., et al. (2023). Detachable MOF-based core/shell nanoreactor for cancer dual-starvation therapy with reversing glucose and glutamine metabolisms. *Small (Weinheim an der Bergstrasse, Germany),**19*(42), e2303253. 10.1002/smll.202303253 37330663 10.1002/smll.202303253

[76] Jiang, Y., Tan, Y., Xiao, K., Li, X., Shao, K., Song, J., et al. (2021). pH-regulating nanoplatform for the “double channel chase” of tumor cells by the synergistic cascade between chlorine treatment and methionine-depletion starvation therapy. *ACS Applied Materials & Interfaces,**13*(46), 54690–54705. 10.1021/acsami.1c14802 34761894 10.1021/acsami.1c14802

[77] Yu, J., Wei, Z., Li, Q., Wan, F., Chao, Z., Zhang, X., et al. (2021). Advanced cancer starvation therapy by simultaneous deprivation of lactate and glucose using a MOF nanoplatform. *Advanced Science (Weinh),**8*(19), e2101467. 10.1002/advs.202101467 10.1002/advs.202101467 PMC8498878 34363341

[78] Tian, H., Zhou, L., Wang, Y., Nice, E. C., Huang, C., & Zhang, H. (2022). A targeted nanomodulator capable of manipulating tumor microenvironment against metastasis. *Journal of Controlled Release,**348*, 590–600. 10.1016/j.jconrel.2022.06.022 35716882 10.1016/j.jconrel.2022.06.022

[79] Yin, J., Wang, X., Sun, X., Dai, H., Song, X., Li, B., et al. (2021). Thrombin based photothermal-responsive nanoplatform for tumor-specific embolization therapy. *Small (Weinheim an der Bergstrasse, Germany),**17*(52), e2105033. 10.1002/smll.202105033 34729905 10.1002/smll.202105033

[80] Liu, X., Zhu, Y.-X., Jia, H.-R., Zhang, X., Gao, G., Xu, K.-F., et al. (2023). A MOF-based nanobomb for tumor microvascular occlusion and combined tumor starvation therapy and chemotherapy. *Materials Today Nano,**24*,. 10.1016/j.mtnano.2023.100430

[81] Shi, R., Liao, C., & Zhang, Q. (2021). Hypoxia-driven effects in cancer: Characterization, mechanisms, and therapeutic implications. *Cells, 10*(3). 10.3390/cells10030678. 10.3390/cells10030678 PMC8003323 33808542

[82] Metkar, S. P., Fernandes, G., Navti, P. D., Nikam, A. N., Kudarha, R., Dhas, N., et al. (2023). Nanoparticle drug delivery systems in hepatocellular carcinoma: A focus on targeting strategies and therapeutic applications. *OpenNano,**12*,. 10.1016/j.onano.2023.100159

[83] Raut, G. K., Chakrabarti, M., Pamarthy, D., & Bhadra, M. P. (2019). Glucose starvation-induced oxidative stress causes mitochondrial dysfunction and apoptosis via Prohibitin 1 upregulation in human breast cancer cells. *Free Radical Biology & Medicine,**145*, 428–441. 10.1016/j.freeradbiomed.2019.09.020 31614178 10.1016/j.freeradbiomed.2019.09.020

[84] Zhang, Y., Wan, Y., Liao, Y., Hu, Y., Jiang, T., He, T., et al. (2020). Janus gamma-Fe(2)O(3)/SiO(2)-based nanotheranostics for dual-modal imaging and enhanced synergistic cancer starvation/chemodynamic therapy. *Science Bulletin (Beijing),**65*(7), 564–572. 10.1016/j.scib.2019.12.024 10.1016/j.scib.2019.12.024 36659188

[85] Zhang, X., He, C., Chen, Y., Chen, C., Yan, R., Fan, T., et al. (2021). Cyclic reactions-mediated self-supply of H2O2 and O2 for cooperative chemodynamic/starvation cancer therapy. *Biomaterials,**275*,. 10.1016/j.biomaterials.2021.120987 10.1016/j.biomaterials.2021.120987 34175561

[86] Ni, W., Jiang, K., Ke, Q., Su, J., Cao, X., Zhang, L., et al. (2023). Development of an intelligent heterojunction fenton catalyst for chemodynamic/starvation synergistic cancer therapy. *Journal of Materials Science & Technology,**141*, 11–20. 10.1016/j.jmst.2022.10.001

[87] Yu, Q., Zhou, J., Song, J., Zhou, H., Kang, B., Chen, H. Y., et al. (2023). A cascade nanoreactor of metal-protein-polyphenol capsule for oxygen-mediated synergistic tumor starvation and chemodynamic therapy. *Small (Weinheim an der Bergstrasse, Germany),**19*(5), e2206592. 10.1002/smll.202206592 36437115 10.1002/smll.202206592

[88] Guo, W., Ren, Y., Chen, Z., Shen, G., Lu, Y., Zhou, H., et al. (2023). Targeted magnetic resonance imaging/near‐infrared dual‐modal imaging and ferroptosis/starvation therapy of gastric cancer with peritoneal metastasis. *Advanced Functional Materials, 33*(27). 10.1002/adfm.202213921.

[89] Wang, Y., Xie, K., Chen, W., Fang, Y., Mo, Q., Zhang, H., et al. (2023). Synergistic ferroptosis-starvation therapy for bladder cancer based on hyaluronic acid modified metal-organic frameworks. *Bioengineering & Translational Medicine,**8*(3), e10515. 10.1002/btm2.10515 37206228 10.1002/btm2.10515 PMC10189452

[90] Wan, X., Song, L., Pan, W., Zhong, H., Li, N., & Tang, B. (2020). Tumor-targeted cascade nanoreactor based on metal-organic frameworks for synergistic ferroptosis-starvation anticancer therapy. *ACS Nano,**14*(9), 11017–11028. 10.1021/acsnano.9b07789 32786253 10.1021/acsnano.9b07789

[91] Ren, Z., Han, X., Wang, L., & Wang, Y. (2022). Hyaluronic acid functionalized ZnO nanoparticles co-deliver AS and GOD for synergistic cancer starvation and oxidative damage. *Science and Reports,**12*(1), 4574. 10.1038/s41598-022-08627-w 10.1038/s41598-022-08627-w PMC8931118 35301389

[92] Zhang, J., Liang, C., Wei, Z., Yang, W., Ge, W., Qu, X., et al. (2022). TME-triggered MnSiO(3)@Met@GOx nanosystem for ATP dual-inhibited starvation/chemodynamic synergistic therapy. *Biomaterials,**287*, 121682. 10.1016/j.biomaterials.2022.121682 35870264 10.1016/j.biomaterials.2022.121682

[93] Wang, J., Yao, L., Hu, E., Cui, Y., Yang, D., & Qian, G. (2021). MnO2 decorated ZIF-8@GOx for synergistic chemodynamic and starvation therapy of cancer. *Journal of Solid State Chemistry,**298*,. 10.1016/j.jssc.2021.122102

[94] Li, C., Wan, Y., Zhang, Y., Fu, L. H., Blum, N. T., Cui, R., et al. (2021). In situ sprayed starvation/chemodynamic therapeutic gel for post‐surgical treatment of IDH1 (R132H) glioma. *Advanced Materials, 34*(5). 10.1002/adma.202103980. 10.1002/adma.202103980 34775641

[95] Wu, H., Li, X., Liu, S., Wang, Q., Cao, Y., Hao, J. N., et al. (2023). GSH-responsive organosilica hybrid nanosystem as a cascade promoter for enhanced starvation and chemodynamic therapy. *Advanced Healthcare Materials,**12*(2), e2201262. 10.1002/adhm.202201262 36213949 10.1002/adhm.202201262

[96] Liu, Y., Chen, K., Yang, Y., & Shi, P. (2023). Glucose oxidase-modified metal-organic framework for starving-enhanced chemodynamic therapy. *ACS Applied Bio Materials,**6*(2), 857–864. 10.1021/acsabm.2c01004 36633432 10.1021/acsabm.2c01004

[97] Li, L., Lin, Z., Xu, X., Wang, W., Chen, H., Feng, Z., et al. (2023). A pH/GSH/glucose responsive nanozyme for tumor cascade amplified starvation and chemodynamic theranostics. *ACS Applied Materials & Interfaces,**15*(35), 41224–41236. 10.1021/acsami.3c05412 37615578 10.1021/acsami.3c05412

[98] Xiao, Y., Lai, F., Xu, M., Zheng, D., Hu, Y., Sun, M., et al. (2023). Dual-functional nanoplatform based on bimetallic metal-organic frameworks for synergistic starvation and chemodynamic therapy. *ACS Biomaterials Science & Engineering,**9*(4), 1991–2000. 10.1021/acsbiomaterials.2c01476 36989499 10.1021/acsbiomaterials.2c01476

[99] Xing, Z., Li, L., Liao, T., Wang, J., Guo, Y., Xu, Z., et al. (2024). A multifunctional cascade enzyme system for enhanced starvation/chemodynamic combination therapy against hypoxic tumors. *Journal of Colloid and Interface Science,**666*, 244–258. 10.1016/j.jcis.2024.04.036 38598997 10.1016/j.jcis.2024.04.036

[100] Huang, Y., Wu, S., Zhang, L., Deng, Q., Ren, J., & Qu, X. (2022). A metabolic multistage glutathione depletion used for tumor-specific chemodynamic therapy. *ACS Nano,**16*(3), 4228–4238. 10.1021/acsnano.1c10231 35213138 10.1021/acsnano.1c10231

[101] Wang, Y., & Song, M. (2020). pH-responsive cascaded nanocatalyst for synergistic like-starvation and chemodynamic therapy. *Colloids and Surfaces. B, Biointerfaces,**192*, 111029. 10.1016/j.colsurfb.2020.111029 32315919 10.1016/j.colsurfb.2020.111029

[102] Xu, M., Liu, Y., Luo, W., Tan, F., Dong, D., Li, W., et al. (2023). A multifunctional nanocatalytic system based on chemodynamic-starvation therapies with enhanced efficacy of cancer treatment. *Journal of Colloid and Interface Science,**630*(Pt B), 804–816. 10.1016/j.jcis.2022.10.145 36356448 10.1016/j.jcis.2022.10.145

[103] Ming, J., Zhu, T., Yang, W., Shi, Y., Huang, D., Li, J., et al. (2020). Pd@Pt-GOx/HA as a novel enzymatic cascade nanoreactor for high-efficiency starving-enhanced chemodynamic cancer therapy. *ACS Applied Materials & Interfaces,**12*(46), 51249–51262. 10.1021/acsami.0c15211 33161703 10.1021/acsami.0c15211

[104] Wang, Z., Liu, B., Sun, Q., Dong, S., Kuang, Y., Dong, Y., et al. (2020). Fusiform-like copper(II)-based metal-organic framework through relief hypoxia and GSH-depletion co-enhanced starvation and chemodynamic synergetic cancer therapy. *ACS Applied Materials & Interfaces,**12*(15), 17254–17267. 10.1021/acsami.0c01539 32227859 10.1021/acsami.0c01539

[105] Moonshi, S. S., Vazquez-Prada, K. X., Adelnia, H., van Holthe, N. J. W., Wu, Y., Tang, J., et al. (2024). Polysuccinimide-based nanoparticle: A nanocarrier with drug release delay and zero burst release properties for effective theranostics of cancer. *Applied Materials Today,**37*, 102150.

[106] Moonshi, S. S., Vazquez-Prada, K. X., Tang, J., Westra van Holthe, N. J., Cowin, G., Wu, Y., et al. (2023). Spiky silver–iron oxide nanohybrid for effective dual-imaging and synergistic thermo-chemotherapy. *ACS Applied Materials & Interfaces,**15*(36), 42153–42169. 37602893 10.1021/acsami.3c04696

[107] Zhang, H., Wu, M., Sumadi, F. A. N., Fu, C., Meng, Q., Alanazi, M., et al. (2024). Responsive theranostic nanoprobe for ratiometric photoacoustic monitoring of hypochlorous acid-mediated inflammation in cancer photothermal therapy. *Advanced Functional Materials,**35*(13), 2414788.

[108] Opoku-Damoah, Y., Zhang, R., Ta, H. T., & Xu, Z. P. (2023). Simultaneous light-triggered release of nitric oxide and carbon monoxide from a lipid-coated upconversion nanosystem inhibits colon tumor growth. *ACS Applied Materials & Interfaces,**15*(49), 56796–56806. 10.1021/acsami.3c13165 38038959

[109] Gao, X., Feng, J., Song, S., Liu, K., Du, K., Zhou, Y., et al. (2022). Tumor-targeted biocatalyst with self-accelerated cascade reactions for enhanced synergistic starvation and photodynamic therapy. *Nano Today,**43*,. 10.1016/j.nantod.2022.101433

[110] Yoo, J. O., & Ha, K. S. (2012). New insights into the mechanisms for photodynamic therapy-induced cancer cell death. *International Review of Cell and Molecular Biology,**295*, 139–174. 10.1016/B978-0-12-394306-4.00010-1 22449489 10.1016/B978-0-12-394306-4.00010-1

[111] Bian, Y., Liu, B., Ding, B., Yuan, M., Yang, C., Li, K., et al. (2024). An enzyme‐engineered coppery nanozyme for high‐efficiency mild photothermal/chemodynamic/starvation therapy through self‐reinforcing cancer energy metabolism regulation. *Advanced Functional Materials, 34*(22). 10.1002/adfm.202313853.

[112] Hu, J. J., Liu, M. D., Gao, F., Chen, Y., Peng, S. Y., Li, Z. H., et al. (2019). Photo-controlled liquid metal nanoparticle-enzyme for starvation/photothermal therapy of tumor by win-win cooperation. *Biomaterials,**217*, 119303. 10.1016/j.biomaterials.2019.119303 31271859 10.1016/j.biomaterials.2019.119303

[113] Wang, W., Yang, Y., Chen, X., Zhao, T., & Li, X. (2023). Hollow mesoporous MnO2 nanospheres as light source-free carriers for synergistic starvation and chemiexcited photodynamic tumor therapy. *ACS Applied Nano Materials,**6*(16), 15314–15323. 10.1021/acsanm.3c03336

[114] Zhang, D. Y., Liang, Y., Wang, M., Younis, M. R., Yi, H., Zhao, X., et al. (2023). Self-assembled carrier-free nanodrugs for starvation therapy-amplified photodynamic therapy of cancer. *Advanced Healthcare Materials,**12*(20), e2203177. 10.1002/adhm.202203177 36947826 10.1002/adhm.202203177

[115] Lv, Z., Jin, L., Gao, W., Cao, Y., Zhang, H., Xue, D., et al. (2022). Novel YOF-based theranostic agents with a cascade effect for NIR-II fluorescence imaging and synergistic starvation/photodynamic therapy of orthotopic gliomas. *ACS Applied Materials & Interfaces,**14*(27), 30523–30532. 10.1021/acsami.2c05354 35775188 10.1021/acsami.2c05354

[116] Zhu, D., Zhang, T., Li, Y., Huang, C., Suo, M., Xia, L., et al. (2022). Tumor-derived exosomes co-delivering aggregation-induced emission luminogens and proton pump inhibitors for tumor glutamine starvation therapy and enhanced type-I photodynamic therapy. *Biomaterials,**283*, 121462. 10.1016/j.biomaterials.2022.121462 35272223 10.1016/j.biomaterials.2022.121462

[117] Wu, X., Fan, Y., Wang, K., Miao, Y., Chang, Y., Ming, J., et al. (2024). NIR-II imaging-guided precise photodynamic therapy for augmenting tumor-starvation therapy by glucose metabolism reprogramming interference. *Science Bulletin (Beijing),**69*(9), 1263–1274. 10.1016/j.scib.2024.02.008 10.1016/j.scib.2024.02.008 38418300

[118] Jiang, R., Hang, L., Li, W., Ling, H., Wang, H., Lei, Q., et al. (2023). Tri-stimulus-responsive hollow mesoporous MnO2 nanocarriers for magnetic-resonance-imaging-guided synergistic starvation/photodynamic therapy of breast cancer. *ACS Applied Nano Materials,**7*(1), 1450–1461. 10.1021/acsanm.3c05733

[119] Zhu, Y., Shi, H., Li, T., Yu, J., Guo, Z., Cheng, J., et al. (2020). A dual functional nanoreactor for synergistic starvation and photodynamic therapy. *ACS Applied Materials & Interfaces,**12*(16), 18309–18318. 10.1021/acsami.0c01039 32233414 10.1021/acsami.0c01039

[120] Ouyang, Y., Wang, P., Huang, B., Yang, G., Tian, J., & Zhang, W. (2021). Zeolitic imidazolate framework platform for combinational starvation therapy and oxygen self-sufficient photodynamic therapy against a hypoxia tumor. *ACS Applied Bio Materials,**4*(5), 4413–4421. 10.1021/acsabm.1c00174 35006853 10.1021/acsabm.1c00174

[121] Li, X., Man, J., Hu, H., Ye, J., & Jin, Q. (2022). Oxygen-economizing liposomes for synergistic photodynamic and starvation therapy. *Colloid and Interface Science Communications,**47*,. 10.1016/j.colcom.2022.100598

[122] Fan, X., Luo, Z., Chen, Y., Yeo, J. C. C., Li, Z., Wu, Y. L., et al. (2022). Oxygen self-supplied enzyme nanogels for tumor targeting with amplified synergistic starvation and photodynamic therapy. *Acta Biomaterialia,**142*, 274–283. 10.1016/j.actbio.2022.01.056 35114372 10.1016/j.actbio.2022.01.056

[123] Li, W., Liu, S., Dong, S., Gai, S., Zhang, F., Dong, Y., et al. (2021). A smart nanoplatform for synergistic starvation, hypoxia-active prodrug treatment and photothermal therapy mediated by near-infrared-II light. *Chemical Engineering Journal,**405*,. 10.1016/j.cej.2020.127027

[124] Dai, Y., Sun, Z., Zhao, H., Qi, D., Li, X., Gao, D., et al. (2021). NIR-II fluorescence imaging guided tumor-specific NIR-II photothermal therapy enhanced by starvation mediated thermal sensitization strategy. *Biomaterials,**275*, 120935. 10.1016/j.biomaterials.2021.120935 34116284 10.1016/j.biomaterials.2021.120935

[125] Song, S., Wang, D., Zhao, K., Wu, Y., Zhang, P., Liu, J., et al. (2022). Donor-acceptor structured photothermal COFs for enhanced starvation therapy. *Chemical Engineering Journal,**442*,. 10.1016/j.cej.2022.135963

[126] Gong, P., Li, C., Wang, D., Song, S., Wu, W., Liu, B., et al. (2023). Enzyme coordination conferring stable monodispersity of diverse metal-organic frameworks for photothermal/starvation therapy. *Journal of Colloid and Interface Science,**642*, 612–622. 10.1016/j.jcis.2023.03.178 37028168 10.1016/j.jcis.2023.03.178

[127] He, X., Hao, Y., Chu, B., Yang, Y., Sun, A., Shi, K., et al. (2021). Redox-­activatable photothermal therapy and enzyme-mediated tumor starvation for synergistic cancer therapy. *Nano Today,**39*,. 10.1016/j.nantod.2021.101174

[128] Zhang, B., Li, X., Shu, W., Yang, Y.-S., Zhu, H.-L., & Shao, C. (2022). A self-supplied O2 versatile nanoplatform for GOx-mediated synergistic starvation and hypothermal photothermal therapy. *Materials & Design,**222*,. 10.1016/j.matdes.2022.111067

[129] Zhang, Y., Wang, K., Xing, G., Dong, X., Zhu, D., Yang, W., et al. (2022). Nanozyme-laden intelligent macrophage EXPRESS amplifying cancer photothermal-starvation therapy by responsive stimulation. *Materials Today Bio,**16*, 100421. 10.1016/j.mtbio.2022.100421 36105675 10.1016/j.mtbio.2022.100421 PMC9464963

[130] Zou, Y., Liu, W., Sun, W., Du, J., Fan, J., & Peng, X. (2022). Highly inoxidizable heptamethine cyanine–glucose oxidase conjugate nanoagent for combination of enhanced photothermal therapy and tumor starvation. *Advanced Functional Materials, 32*(17). 10.1002/adfm.202111853.

[131] Li, X., Wu, H., Jiang, C., Zou, J., Wang, Q., Guan, M., et al. (2022). Engineered organosilica hybrid micelles for photothermal-enhanced starvation cancer therapy. *Chemistry - An Asian Journal,**17*(17), e202200570. 10.1002/asia.202200570 35785417 10.1002/asia.202200570

[132] Duan, X., Tian, H., Zheng, S., Zhu, J., Li, C., He, B., et al. (2023). Photothermal-starvation therapy nanomodulator capable of inhibiting colorectal cancer recurrence and metastasis by energy metabolism reduction. *Advanced Healthcare Materials,**12*(26), e2300968. 10.1002/adhm.202300968 37543843 10.1002/adhm.202300968

[133] Zhang, W. X., Zhou, Z. L., Lv, Q. Y., Song, X., Chen, J., Niu, C. B., et al. (2023). O(2)-generation-enhanced responsive starvation/photothermal synergistic tumor therapy based on the AuNRs@MnO(2)@SiO(2) nanocarrier and thermosensitive biomimetic camouflaging. *ACS Applied Bio Materials,**6*(11), 4775–4790. 10.1021/acsabm.3c00544 37830366 10.1021/acsabm.3c00544

[134] Wu, C. Y., Hsu, Y. H., Chen, Y., Yang, L. C., Tseng, S. C., Chen, W. R., et al. (2021). Robust O(2) supplementation from a trimetallic nanozyme-based self-sufficient complementary system synergistically enhances the starvation/photothermal therapy against hypoxic tumors. *ACS Applied Materials & Interfaces,**13*(32), 38090–38104. 10.1021/acsami.1c10656 34342219 10.1021/acsami.1c10656

[135] Zhuang, J., Qin, Y. T., Feng, Y. S., Su, Z. C., He, X. W., Li, W. Y., et al. (2023). A novel hexokinases inhibitor based on molecularly imprinted polymer for combined starvation and enhanced photothermal therapy of malignant tumors. *ACS Applied Materials & Interfaces,**15*(21), 25898–25908. 10.1021/acsami.3c00079 37191997 10.1021/acsami.3c00079

[136] Li, M., Li, N., Qi, J., Gao, D., Zhou, M., Wei, X., et al. (2022). Mild-temperature photothermal effect enhanced by functional conjugated polymer nanoparticles through enzyme-mediated starvation. *ACS Applied Bio Materials,**5*(6), 2536–2542. 10.1021/acsabm.2c00288 35535955 10.1021/acsabm.2c00288

[137] Zhu, H., Li, Y., Ming, Z., & Liu, W. (2021). Glucose oxidase-mediated tumor starvation therapy combined with photothermal therapy for colon cancer. *Biomaterials Science,**9*(16), 5577–5587. 10.1039/d1bm00869b 34241605 10.1039/d1bm00869b

[138] Huis In 't Veld, R. V., Heuts, J., Ma, S., Cruz, L. J., Ossendorp, F. A., & Jager, M. J. (2023). Current challenges and opportunities of photodynamic therapy against cancer. *Pharmaceutics, 15*(2). 10.3390/pharmaceutics15020330. 10.3390/pharmaceutics15020330 PMC9965442 36839652

[139] Han, H. S., & Choi, K. Y. (2021). Advances in nanomaterial-mediated photothermal cancer therapies: Toward clinical applications. *Biomedicines, 9*(3). 10.3390/biomedicines9030305. 10.3390/biomedicines9030305 PMC8002224 33809691

[140] Ding, X., Zang, M., Zhang, Y., Chen, Y., Du, J., Yan, A., et al. (2023). A bioresponsive diselenide-functionalized hydrogel with cascade catalytic activities for enhanced local starvation- and hypoxia-activated melanoma therapy. *Acta Biomaterialia,**167*, 182–194. 10.1016/j.actbio.2023.06.017 37339693 10.1016/j.actbio.2023.06.017

[141] Liu, Y., Guo, K., Ding, M., Zhang, B., Xiao, N., Tang, Z., et al. (2022). Engineered magnetic polymer nanoparticles can ameliorate breast cancer treatment inducing pyroptosis-starvation along with chemotherapy. *ACS Applied Materials & Interfaces,**14*(37), 42541–42557. 10.1021/acsami.2c13011 36094305 10.1021/acsami.2c13011

[142] Hu, J., Hu, J., Wu, W., Qin, Y., Fu, J., Zhou, J., et al. (2022). N-acetyl-galactosamine modified metal-organic frameworks to inhibit the growth and pulmonary metastasis of liver cancer stem cells through targeted chemotherapy and starvation therapy. *Acta Biomaterialia,**151*, 588–599. 10.1016/j.actbio.2022.08.027 36002126 10.1016/j.actbio.2022.08.027

[143] Cai, X., Shi, S., Chen, G., Zhong, M., Yang, Y., Mai, Z., et al. (2023). Glutamine metabolism targeting liposomes for synergistic chemosensitization and starvation therapy in ovarian cancer. *Acta Biomaterialia,**158*, 560–570. 10.1016/j.actbio.2022.12.052 36596434 10.1016/j.actbio.2022.12.052

[144] Shao, F., Wu, Y., Tian, Z., & Liu, S. (2021). Biomimetic nanoreactor for targeted cancer starvation therapy and cascade amplificated chemotherapy. *Biomaterials,**274*, 120869. 10.1016/j.biomaterials.2021.120869 33984636 10.1016/j.biomaterials.2021.120869

[145] Ullah, A., Khan, M., Yibang, Z., Raza, F., Hasnat, M., Cao, J., et al. (2023). Hollow mesoporous silica nanoparticles for dual chemo-starvation therapy of hepatocellular carcinoma. *Pharmaceutical Research,**40*(9), 2215–2228. 10.1007/s11095-023-03599-6 37700104 10.1007/s11095-023-03599-6

[146] Ding, J., Liu, Y., Liu, Z., Tan, J., Xu, W., Huang, G., et al. (2024). Glutathione-responsive organosilica hybrid nanosystems for targeted dual-starvation therapy in luminal breast cancer. *Molecular Pharmaceutics,**21*(2), 745–759. 10.1021/acs.molpharmaceut.3c00894 38148514 10.1021/acs.molpharmaceut.3c00894

[147] Ghosh, A., Ghosh, A. K., Chowdhury, M., & Das, P. K. (2022). Folic acid-functionalized carbon dot-enabled starvation therapy in synergism with paclitaxel against breast cancer. *ACS Applied Bio Materials,**5*(5), 2389–2402. 10.1021/acsabm.2c00235 35452214 10.1021/acsabm.2c00235

[148] Yu, J., He, X., Wang, Z., Liu, S., Hao, D., Li, X., et al. (2021). Combination of starvation therapy and Pt-NP based chemotherapy for synergistic cancer treatment. *Journal of Materials Chemistry B,**9*(32), 6406–6411. 10.1039/d1tb01222c 34318860 10.1039/d1tb01222c

[149] Zhang, Q., Xuan, Q., Wang, C., Shi, C., Wang, X., Ma, T., et al. (2023). Bioengineered “molecular glue”-mediated tumor-specific cascade nanoreactors with self-destruction ability for enhanced precise starvation/chemosynergistic tumor therapy. *ACS Applied Materials & Interfaces,**15*(35), 41271–41286. 10.1021/acsami.3c06871 37622208 10.1021/acsami.3c06871

[150] Zhang, Y., Li, Y., Gao, Z., Ding, B., An, P., Zhang, X., et al. (2020). Mesoporous silica-coated silver nanoframes as drug-delivery vehicles for chemo/starvation/metal ion multimodality therapy. *Langmuir,**36*(23), 6345–6351. 10.1021/acs.langmuir.0c00191 32388995 10.1021/acs.langmuir.0c00191

[151] Gong, P., Zhao, K., Liu, X., Li, C., Liu, B., Hu, L., et al. (2022). Fluorescent COFs with a highly conjugated structure for combined starvation and gas therapy. *ACS Applied Materials & Interfaces,**14*(41), 46201–46211. 10.1021/acsami.2c11423 36208197 10.1021/acsami.2c11423

[152] Peng, J., Gong, P., Song, S., Zhao, K., Zheng, X., Liu, J., et al. (2021). Biomineralized synthesis of a smart O(2)-regenerating nanoreactor for highly efficient starvation/gas therapy. *Materials Science & Engineering, C: Materials for Biological Applications,**126*, 112132. 10.1016/j.msec.2021.112132 34082949 10.1016/j.msec.2021.112132

[153] Zhai, M., Gong, P., Li, H., Peng, J., Xu, W., Song, S., et al. (2021). Metastable interface biomimetic synthesis of a smart nanosystem for enhanced starvation/gas therapy. *Journal of Colloid and Interface Science,**599*, 149–157. 10.1016/j.jcis.2021.04.042 33940438 10.1016/j.jcis.2021.04.042

[154] Liu, W., Semcheddine, F., Guo, Z., Jiang, H., & Wang, X. (2022). Glucose-responsive ZIF-8 nanocomposites for targeted cancer therapy through combining starvation with stimulus-responsive nitric oxide synergistic treatment. *ACS Applied Bio Materials,**5*(6), 2902–2912. 10.1021/acsabm.2c00262 35533346 10.1021/acsabm.2c00262

[155] Fan, X., Chen, B., Xu, H., Pan, A., Liang, S., Tan, S., et al. (2023). Glutathione/glucose-depleting nanoparticles with NO generation for ferroptosis/starvation/NO-induced cancer therapy. *Chemistry of Materials,**35*(8), 3124–3137. 10.1021/acs.chemmater.2c03612

[156] Opoku-Damoah, Y., Xu, Z. P., Ta, H. T., & Zhang, R. (2024). Ultrasound-responsive lipid nanoplatform with nitric oxide and carbon monoxide release for cancer sono-gaso-therapy. *ACS Applied Bio Materials,**7*(11), 7585–7594. 39509170 10.1021/acsabm.4c01165

[157] Zhao, Y., Liu, J., He, M., Dong, Q., Zhang, L., Xu, Z., et al. (2022). Platinum-titania Schottky junction as nanosonosensitizer, glucose scavenger, and tumor microenvironment-modulator for promoted cancer treatment. *ACS Nano,**16*(8), 12118–12133. 10.1021/acsnano.2c02540 35904186 10.1021/acsnano.2c02540

[158] Wang, L., Song, W., Choi, S., Yu, K., Zhang, F., Guo, W., et al. (2023). Hollow CoP@N-carbon nanospheres: Heterostructure and glucose-enhanced charge separation for sonodynamic/starvation therapy. *ACS Applied Materials & Interfaces,**15*(2), 2552–2563. 10.1021/acsami.2c15327 36600575 10.1021/acsami.2c15327

[159] Bao, Y., Chen, J., Qiu, H., Zhang, C., Huang, P., Mao, Z., et al. (2021). Erythrocyte membrane-camouflaged PCN-224 nanocarriers integrated with platinum nanoparticles and glucose oxidase for enhanced tumor sonodynamic therapy and synergistic starvation therapy. *ACS Applied Materials & Interfaces,**13*(21), 24532–24542. 10.1021/acsami.1c05644 34019368 10.1021/acsami.1c05644

[160] Huang, L., Su, Y., Hu, X., Zhang, Y., Xu, G., Chen, S., et al. (2024). An ultrasound-activated nanozyme sonosensitizer for photoacoustic imaging-guided breast cancer sonodynamic and starvation combination therapy. *ACS Applied Nano Materials,**7*(4), 4441–4452. 10.1021/acsanm.3c05959

[161] Wang, H., Li, D., Wang, H., Ren, Q., Pan, Y., Dao, A., et al. (2024). Enhanced sonodynamic therapy for deep tumors using a self-assembled organoplatinum(II) sonosensitizer. *Journal of Medicinal Chemistry,**67*(20), 18356–18367. 10.1021/acs.jmedchem.4c01671 39360515 10.1021/acs.jmedchem.4c01671

[162] Zhao, M., Zhu, A., Zheng, X., Qian, X., Zhang, S., Wu, C., et al. (2023). Multistage-responsive dual-enzyme nanocascades for synergistic radiosensitization-starvation cancer therapy. *Advanced Healthcare Materials,**12*(21), e2300118. 10.1002/adhm.202300118 37094801 10.1002/adhm.202300118

[163] Fan, R., Chen, C., Hu, J., Mu, M., Chuan, D., Chen, Z., et al. (2023). Multifunctional gold nanorods in low-temperature photothermal interactions for combined tumor starvation and RNA interference therapy. *Acta Biomaterialia,**159*, 324–337. 10.1016/j.actbio.2023.01.036 36706851 10.1016/j.actbio.2023.01.036

[164] Lu, Z., Gao, J., Fang, C., Zhou, Y., Li, X., & Han, G. (2020). Porous Pt nanospheres incorporated with GOx to enable synergistic oxygen-inductive starvation/electrodynamic tumor therapy. *Advanced Science (Weinh),**7*(17), 2001223. 10.1002/advs.202001223 10.1002/advs.202001223 PMC7507307 32995127

[165] Dai, L., Yao, M., Fu, Z., Li, X., Zheng, X., Meng, S., et al. (2022). Multifunctional metal-organic framework-based nanoreactor for starvation/oxidation improved indoleamine 2,3-dioxygenase-blockade tumor immunotherapy. *Nature Communications,**13*(1), 2688. 10.1038/s41467-022-30436-y 35577812 10.1038/s41467-022-30436-y PMC9110376

[166] Zhang, C., Han, Z. Y., Chen, K. W., Wang, Y. Z., Bao, P., Ji, P., et al. (2024). In situ formed microalgae-integrated living hydrogel for enhanced tumor starvation therapy and immunotherapy through photosynthetic oxygenation. *Nano Letters,**24*(12), 3801–3810. 10.1021/acs.nanolett.4c00471 38477714 10.1021/acs.nanolett.4c00471

[167] Kang, H., Chen, L., Li, Q., Chen, H., & Zhang, L. (2023). Dual-oxygenation/dual-fenton synergistic photothermal/chemodynamic/starvation therapy for tumor treatment. *ACS Applied Materials & Interfaces,**15*(12), 15129–15139. 10.1021/acsami.2c22578 36919267 10.1021/acsami.2c22578

[168] Liang, J., Sun, Y., Wang, K., Zhang, Y., Guo, L., Bao, Z., et al. (2023). Prussian blue-derived nanoplatform for in situ amplified photothermal/chemodynamic/starvation therapy. *ACS Applied Materials & Interfaces,**15*(14), 18191–18204. 10.1021/acsami.2c22448 36975190 10.1021/acsami.2c22448

[169] Wu, F., Zhang, Q., Zhang, M., Sun, B., She, Z., Ge, M., et al. (2020). Hollow porous carbon coated FeS(2)-based nanocatalysts for multimodal imaging-guided photothermal, starvation, and triple-enhanced chemodynamic therapy of cancer. *ACS Applied Materials & Interfaces,**12*(9), 10142–10155. 10.1021/acsami.0c00170 32043350 10.1021/acsami.0c00170

[170] Wu, F., Huang, C., Sun, B., Zhu, Z., Cheng, W., Chen, Y., et al. (2022). H2O2 self-supplementing and GSH-depleting nanoreactors based on MoO3–x@Fe3O4-GOD-PVP for photothermally reinforced nanocatalytic cancer therapy at the second near-infrared biowindow. *ACS Sustainable Chemistry & Engineering,**10*(19), 6346–6357. 10.1021/acssuschemeng.2c00964

[171] Singh, P., Youden, B., Yang, Y., Chen, Y., Carrier, A., Cui, S., et al. (2021). Synergistic multimodal cancer therapy using glucose oxidase@CuS nanocomposites. *ACS Applied Materials & Interfaces,**13*(35), 41464–41472. 10.1021/acsami.1c12235 34448397 10.1021/acsami.1c12235

[172] Qiu, S., Wu, X., Li, Z., Xu, X., Wang, J., Du, Y., et al. (2022). A smart nanoreactor based on an O(2)-economized dual energy inhibition strategy armed with dual multi-stimuli-responsive “doorkeepers” for enhanced CDT/PTT of rheumatoid arthritis. *ACS Nano,**16*(10), 17062–17079. 10.1021/acsnano.2c07338 36153988 10.1021/acsnano.2c07338

[173] Zhang, L., Fu, J. M., Song, L. B., Cheng, K., Zhang, F., Tan, W. H., et al. (2024). Ultrasmall Bi/Cu coordination polymer combined with glucose oxidase for tumor enhanced chemodynamic therapy by starvation and photothermal treatment. *Advanced Healthcare Materials,**13*(2), e2302264. 10.1002/adhm.202302264 37812564 10.1002/adhm.202302264

[174] Rao, Y., Fan, T., Zhou, L., Fang, K., Sun, Y., Hu, X., et al. (2023). A positive self-amplified H(2)O(2) and acidity circulation for boosting CDT-PTT-starvation therapy. *Journal of Controlled Release,**354*, 701–712. 10.1016/j.jconrel.2023.01.053 36690036 10.1016/j.jconrel.2023.01.053

[175] Zhou, L., Zhao, J., Chen, Y., Zheng, Y., Li, J., Zhao, J., et al. (2020). MoS(2)-ALG-Fe/GOx hydrogel with Fenton catalytic activity for combined cancer photothermal, starvation, and chemodynamic therapy. *Colloids and Surfaces. B, Biointerfaces,**195*, 111243. 10.1016/j.colsurfb.2020.111243 32663712 10.1016/j.colsurfb.2020.111243

[176] Xu, R., Zhang, D., Tan, J., Ge, N., Liu, D., Liu, J., et al. (2022). A multifunctional cascade bioreactor based on a layered double oxides composite hydrogel for synergetic tumor chemodynamic/starvation/photothermal therapy. *Acta Biomaterialia,**153*, 494–504. 10.1016/j.actbio.2022.09.024 36115653 10.1016/j.actbio.2022.09.024

[177] Ding, M., Kong, X., Chen, W., Yan, L., Huang, H., Lv, Z., et al. (2022). Efficient starvation therapy with three-pathway blocking in combination with PTT/CDT for TME reversal and tumor apoptosis. *Journal of Industrial and Engineering Chemistry,**110*, 456–470. 10.1016/j.jiec.2022.03.022

[178] Wang, M., Chang, M., Zheng, P., Sun, Q., Wang, G., Lin, J., et al. (2022). A noble AuPtAg-GOx nanozyme for synergistic tumor immunotherapy induced by starvation therapy-augmented mild photothermal therapy. *Advanced Science (Weinh),**9*(31), e2202332. 10.1002/advs.202202332 10.1002/advs.202202332 PMC9631081 36156451

[179] Wang, Q., Niu, D., Shi, J., & Wang, L. (2021). A three-in-one ZIFs-derived CuCo(O)/GOx@PCNs hybrid cascade nanozyme for immunotherapy/enhanced starvation/photothermal therapy. *ACS Applied Materials & Interfaces,**13*(10), 11683–11695. 10.1021/acsami.1c01006 33656325 10.1021/acsami.1c01006

[180] Pu, Y., Wu, W., Zhou, B., Xiang, H., Yu, J., Yin, H., et al. (2022). Starvation therapy enabled “switch-on” NIR-II photothermal nanoagent for synergistic in situ photothermal immunotherapy. *Nano Today,**44*,. 10.1016/j.nantod.2022.101461

[181] Li, Z., & Rong, L. (2021). A homotypic membrane-camouflaged biomimetic nanoplatform with gold nanocrystals for synergistic photothermal/starvation/immunotherapy. *ACS Applied Materials & Interfaces,**13*(20), 23469–23480. 10.1021/acsami.1c04305 33999610 10.1021/acsami.1c04305

[182] Zhang, Y., Ouyang, Z., Zhan, M., Yang, R., Gao, Y., Li, L., et al. (2023). An intelligent vascular disrupting dendritic nanodevice incorporating copper sulfide nanoparticles for immune modulation-mediated combination tumor therapy. *Small (Weinheim an der Bergstrasse, Germany),**19*(39), e2301914. 10.1002/smll.202301914 37259269 10.1002/smll.202301914

[183] Zhou, Z., Huang, J., Zhang, Z., Zhang, L., Cao, Y., Xu, Z., et al. (2022). Bimetallic PdPt-based nanocatalysts for photothermal-augmented tumor starvation and sonodynamic therapy in NIR-II biowindow assisted by an oxygen self-supply strategy. *Chemical Engineering Journal,**435*,. 10.1016/j.cej.2022.135085

[184] Du, X., Zhang, Y., Zhang, Y., Gao, S., Yang, X., Ye, L., et al. (2022). Cancer cell membrane camouflaged biomimetic nanosheets for enhanced chemo-photothermal-starvation therapy and tumor microenvironment remodeling. *Applied Materials Today,**29*,. 10.1016/j.apmt.2022.101677

[185] Liu, K., Yan, S., Liu, Z., Wang, D., Yang, Q., Jiang, X., et al. (2022). New anti-tumor strategy based on acid-triggered self-destructive and near-infrared laser light responses of nano-biocatalysts integrating starvation–chemo–photothermal therapies. *Cancer Nanotechnology, 13*(1). 10.1186/s12645-022-00117-y.

[186] Sun, P., Yang, W., He, J., He, L., Chen, P., Xu, W., et al. (2023). Phenylboronic acid-modified near-infrared region II excitation donor-acceptor-donor molecule for 2-deoxy-d-glucose improved starvation/chemo/photothermal combination therapy. *Advanced Healthcare Materials,**12*(30), e2302099. 10.1002/adhm.202302099 37666241 10.1002/adhm.202302099

[187] Lu, J., Liu, F., Li, H., Xu, Y., & Sun, S. (2020). Width-consistent mesoporous silica nanorods with a precisely controlled aspect ratio for lysosome dysfunctional synergistic chemotherapy/photothermal therapy/starvation therapy/oxidative therapy. *ACS Applied Materials & Interfaces,**12*(22), 24611–24622. 10.1021/acsami.0c06117 32379418 10.1021/acsami.0c06117

[188] Chen, X., Ma, R., Fu, Z., Su, Q., Luo, X., Han, Y., et al. (2022). Metal-phenolic networks-encapsulated cascade amplification delivery nanoparticles overcoming cancer drug resistance via combined starvation/chemodynamic/chemo therapy. *Chemical Engineering Journal,**442*,. 10.1016/j.cej.2022.136221

[189] Guo, Y., Jia, H. R., Zhang, X., Zhang, X., Sun, Q., Wang, S. Z., et al. (2020). A glucose/oxygen-exhausting nanoreactor for starvation- and hypoxia-activated sustainable and cascade chemo-chemodynamic therapy. *Small (Weinheim an der Bergstrasse, Germany),**16*(31), e2000897. 10.1002/smll.202000897 32537936 10.1002/smll.202000897

[190] Peng, H., Qin, Y. T., Feng, Y. S., He, X. W., Li, W. Y., & Zhang, Y. K. (2021). Phosphate-degradable nanoparticles based on metal-organic frameworks for chemo-starvation-chemodynamic synergistic antitumor therapy. *ACS Applied Materials & Interfaces,**13*(31), 37713–37723. 10.1021/acsami.1c10816 34340302 10.1021/acsami.1c10816

[191] Chen, W. J., Gupta, D., Yang, M., Yang, F., Feng, N., Song, J., et al. (2023). A purposefully designed ph/gsh-responsive mnfe-based metal-organic frameworks as cascade nanoreactor for enhanced chemo-chemodynamic-starvation synergistic therapy. *Small (Weinheim an der Bergstrasse, Germany),**19*(50), e2303403. 10.1002/smll.202303403 37649230 10.1002/smll.202303403

[192] Bian, Y., Liu, B., Ding, B., Wang, M., Yuan, M., Ma, P., et al. (2023). Tumor microenvironment-activated nanocomposite for self-amplifying chemodynamic/starvation therapy enhanced IDO-blockade tumor immunotherapy. *Advanced Science (Weinh),**10*(34), e2303580. 10.1002/advs.202303580 10.1002/advs.202303580 PMC10700178 37807763

[193] Gao, Y., Ouyang, Z., Shen, S., Yu, H., Jia, B., Wang, H., et al. (2023). Manganese dioxide-entrapping dendrimers co-deliver protein and nucleotide for magnetic resonance imaging-guided chemodynamic/starvation/immune therapy of tumors. *ACS Nano,**17*(23), 23889–23902. 10.1021/acsnano.3c08174 38006397 10.1021/acsnano.3c08174

[194] Hao, Z., Cheng, X., Cong, C., Zhang, X., Zhang, W., Zhao, Q., et al. (2021). Nanoreactor of “butterfly effect” inciting a triple interlocked combination of starvation/chemo/metal ion therapy by remodeling tumor microenvironment. *Chemical Engineering Journal,**405*,. 10.1016/j.cej.2020.126571

[195] Hu, T., Jia, L., Li, H., Yang, C., Yan, Y., Lin, H., et al. (2024). An intelligent and soluble microneedle composed of Bi/BiVO(4) Schottky heterojunction for tumor ct imaging and starvation/gas therapy-promoted synergistic cancer treatment. *Advanced Healthcare Materials,**13*(8), e2303147. 10.1002/adhm.202303147 38206853 10.1002/adhm.202303147

[196] Zhu, J., Wang, C., Wei, Q., Su, Y., Qu, X., Wang, W., et al. (2023). PtMo-Au metalloenzymes regulated tumor microenvironment for enhanced sonodynamic/chemodynamic/starvation synergistic therapy. *Small (Weinheim an der Bergstrasse, Germany),**19*(45), Article e2303365. 10.1002/smll.202303365 37431203 10.1002/smll.202303365

[197] Zhang, J., Yang, J., Qin, X., Zhuang, J., Jing, D., Ding, Y., et al. (2022). Glucose oxidase integrated porphyrinic covalent organic polymers for combined photodynamic/chemodynamic/starvation therapy in cancer treatment. *ACS Biomaterials Science & Engineering,**8*(5), 1956–1963. 10.1021/acsbiomaterials.2c00138 35412788 10.1021/acsbiomaterials.2c00138

[198] Chen, M., Yang, Y., Tang, L., He, S., Guo, W., Ge, G., et al. (2023). Iron-rich semiconducting polymer dots for the combination of ferroptosis-starvation and phototherapeutic cancer therapy. *Advanced Healthcare Materials,**12*(26), e2300839. 10.1002/adhm.202300839 37354132 10.1002/adhm.202300839

[199] Sungu Akdogan, C. Z., Akbay Cetin, E., Onur, M. A., Onel, S., & Tuncel, A. (2024). In vitro synergistic photodynamic, photothermal, chemodynamic, and starvation therapy performance of chlorin e6 immobilized, polydopamine-coated hollow, porous ceria-based, hypoxia-tolerant nanozymes carrying a cascade system. *ACS Applied Bio Materials,**7*(5), 2781–2793. 10.1021/acsabm.3c01181 38380497 10.1021/acsabm.3c01181 PMC11110068

[200] Sun, R., Ge, Y., Liu, H., He, P., Song, W., & Zhang, X. (2021). Erythrocyte membrane-encapsulated glucose oxidase and manganese/ferrite nanocomposite as a biomimetic “all in one” nanoplatform for cancer therapy. *ACS Applied Bio Materials,**4*(1), 701–710. 10.1021/acsabm.0c01226

[201] Ciou, T. Y., Korupalli, C., Chou, T. H., Hsiao, C. H., Getachew, G., Bela, S., et al. (2021). Biomimetic nanoreactor for cancer eradication via win-win cooperation between starvation/photo/chemodynamic therapies. *ACS Applied Bio Materials,**4*(7), 5650–5660. 10.1021/acsabm.1c00452 35006729 10.1021/acsabm.1c00452

[202] Cao, Y., Jin, L., Zhang, S., Lv, Z., Yin, N., Zhang, H., et al. (2023). Blood-brain barrier permeable and multi-stimuli responsive nanoplatform for orthotopic glioma inhibition by synergistic enhanced chemo-/chemodynamic/photothermal/starvation therapy. *European Journal of Pharmaceutical Sciences,**180*, 106319. 10.1016/j.ejps.2022.106319 36328086 10.1016/j.ejps.2022.106319

[203] He, Y., Pan, Y., Zhao, X., Ye, L., Liu, L., Wang, W., et al. (2023). Camouflaging multifunctional nanoparticles with bacterial outer membrane for augmented chemodynamic/photothermal/starvation/chemo multimodal synergistic therapy of orthotopic glioblastoma. *Chemical Engineering Journal,**471*,. 10.1016/j.cej.2023.144410

[204] Zhang, Y., Yang, Y., Shi, J., & Wang, L. (2021). A multimodal strategy of Fe(3)O(4)@ZIF-8/GOx@MnO(2) hybrid nanozyme via TME modulation for tumor therapy. *Nanoscale,**13*(39), 16571–16588. 10.1039/d1nr04196g 34585187 10.1039/d1nr04196g

[205] Meng, Y., Yang, D., Yan, Y., Yang, C., Yang, Z., & Guo, W. (2024). An injectable hydrogel based on MOF-derived hollow nanocomposites for starvation and gas therapy cooperated phototherapy. *Materials Letters,**357*,. 10.1016/j.matlet.2023.135639

[206] Li, G., Lu, X., Zhang, S., Zhang, J., Fu, X., Zhang, M., et al. (2023). Multi-enzyme cascade-triggered nitric oxide release nanoplatform combined with chemo starvation-like therapy for multidrug-resistant cancers. *ACS Applied Materials & Interfaces,**15*(26), 31285–31299. 10.1021/acsami.3c05337 37344958 10.1021/acsami.3c05337

[207] Luo, Y., Zhang, L., Wang, S., Wang, Y., Hua, J., Wen, C., et al. (2023). H(2)O(2) self-supply and glutathione depletion engineering nanoassemblies for NIR-II photoacoustic imaging of tumor tissues and photothermal-enhanced gas starvation-primed chemodynamic therapy. *ACS Applied Materials & Interfaces,**15*(32), 38309–38322. 10.1021/acsami.3c07227 37534669 10.1021/acsami.3c07227

[208] Wisniewska, J., Sobczak, I., & Ziolek, M. (2021). Gold based on SBA-15 supports – Promising catalysts in base-free glucose oxidation. *Chemical Engineering Journal,**413*,. 10.1016/j.cej.2020.127548

[209] Yuan, F., Li, Z. D., Li, Q., Zeng, Y., Zhang, G., & Li, Y. (2025). Designing nanoparticles to minimize unintended inflammatory responses: A step toward safer and more effective precision nanomedicine. *Nanomedicine (Lond),**1–5*,. 10.1080/17435889.2025.2476377 10.1080/17435889.2025.2476377 PMC12140478 40066498

